# Tissue and Process Specific microRNA–mRNA Co-Expression in Mammalian Development and Malignancy

**DOI:** 10.1371/journal.pone.0005436

**Published:** 2009-05-05

**Authors:** Hongye Liu, Issac S. Kohane

**Affiliations:** 1 Informatics Program, Children's Hospital, Boston, Massachusetts, United States of America; 2 Cancer Biology, Dana Farber Cancer Institute, Boston, Massachusetts, United States of America; 3 Partners Center for Genetics and Genomics, Harvard Medical School, Boston, Massachusetts, United States of America; 4 Division of Health Sciences and Technology, Harvard University and Massachusetts Institute of Technology, Cambridge, Massachusetts, United States of America; University of Calgary, Canada

## Abstract

An association between enrichment and depletion of microRNA (miRNA) binding sites, 3′ UTR length, and mRNA expression has been demonstrated in various developing tissues and tissues from different mature organs; but functional, context-dependent miRNA regulations have yet to be elucidated. Towards that goal, we examined miRNA–mRNA interactions by measuring miRNA and mRNA in the same tissue during development and also in malignant conditions. We identified significant miRNA-mediated biological process categories in developing mouse cerebellum and lung using non-targeted mRNA expression as the negative control. Although miRNAs in general suppress target mRNA messages, many predicted miRNA targets demonstrate a significantly higher level of co-expression than non-target genes in developing cerebellum. This phenomenon is tissue specific since it is not observed in developing lungs. Comparison of mouse cerebellar development and medulloblastoma demonstrates a shared miRNA–mRNA co-expression program for brain-specific neurologic processes such as *synaptic transmission* and *exocytosis*, in which miRNA target expression increases with the accumulation of multiple miRNAs in developing cerebellum and decreases with the loss of these miRNAs in brain tumors. These findings demonstrate the context-dependence of miRNA–mRNA co-expression.

## Introduction

MicroRNAs (miRNA) are short (∼22 nt), single-stranded non-coding RNAs that regulate mRNA gene expression at multiple levels [1]–[6]. The importance of these micro-regulators is evidenced by the increasing number of miRNAs that have been identified; up to 1/3 of human genes are estimated to be miRNA targets. Detailed studies of the expression of both individual miRNAs [7]–[14] and large sets of miRNAs [2], [15]–[16] indicate that, in general, miRNAs suppress mRNA messages. In studies of the expression of large miRNA sets, enrichment or depletion of miRNA binding sites and 3′ UTR length have been evaluated with respect to gene expression in various tissues and during development. Farh et al. reported miRNA-induced repression of mRNA in myoblast differentiation and tissue-specific signatures based on comparisons of conserved and non-conserved sites [2]. Stark et al. reported depletion of miRNA binding sites on genes involved in basic cellular processes [16]. For several miRNAs, co-expressed genes avoid miRNA binding sites while target genes and miRNAs are preferentially expressed in neighboring tissues during Drosophila embryonic development. Both Stark et al. and Sood et al. reported a bias of a longer 3′ UTR length and more miRNA binding sites in genes involved in neurogenesis and in genes highly expressed in neuronal tissues [15]–[16].

Although there are tissue-specific signatures of miRNA repression or miRNA–mRNA mutual-exclusiveness for several highly expressed miRNAs, the pattern of miRNA target gene expression is complicated, especially in the central nervous system (CNS) [2], [15]–[16]. We examined miRNA–mRNA interactions by studying large numbers of miRNAs and the expression of their predicted mRNA targets during the same developmental stages in mouse cerebellum as studied in previous reports for the following reasons. First, we wished to capture more than the dependencies/effects of highly expressed miRNAs. Second, the results of biochemical studies indicate that miRNA repression of mRNA is dependent on the specific cellular conditions [17], hence both tissue-specific and temporal-specific studies are needed to define each condition. Third, the extensive transcriptional program of development is well suited for identifying dynamic miRNA/mRNA interactions *in vivo*.

To understand the functional roles of miRNAs during development, we assigned their respective target genes to ontological groups based on Gene Ontology (GO), as described in Sood et al.[15]. For consistency, in this manuscript we used the term “target” for any predicted mRNA gene of some known miRNA, accordingly “non-target” is used for the complement of predicted targets. For each miRNA, we identified the statistically significant GO terms among the miRNA's computationally predicted mRNA “target set” that were differentiated from the non-target genes that had positive-correlated developmental profile to the target set. This comparison with non-target genes was performed because the use of non-target genes as a negative control might allow for better recognition of miRNA-mediated features and minimizes the influence of cell type. We defined a **developmentally** **coherent target [coherent target]** of a miRNA as a predicted target whose expression negatively correlated with the miRNA. The assumption here is that miRNAs primarily act as suppressors of mRNA during development. Accordingly, a **developmentally non-coherent target** [**non-coherent target**] was defined as one whose gene expression was not altered in response to the suppressive function of the miRNA in developing cerebellum. This notion of a non-coherent target is unrelated to Stark's notion of a depletion of miRNA binding sites on mRNA that are co-expressed in a given tissue with an miRNA. Non-coherent targets may co-express with miRNAs despite their 3′UTRs being enriched for the binding sites of those miRNAs.

**Figure 1:**
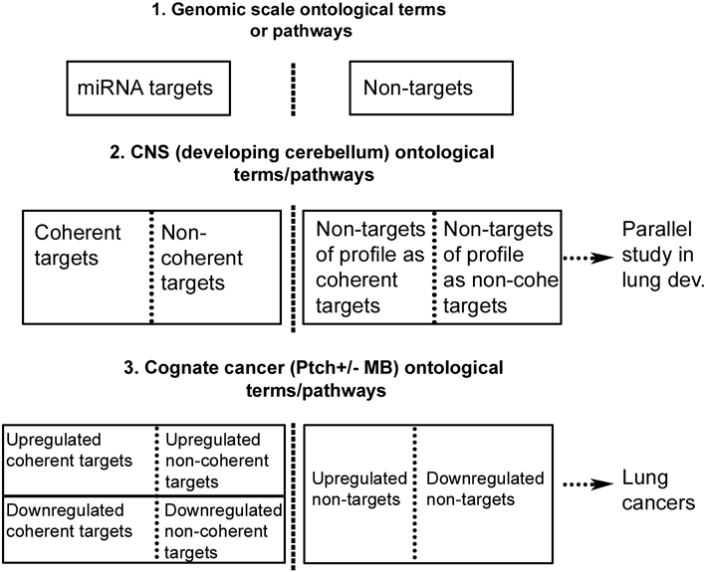
Design flow of the functional tissue-specific study of miRNA–mRNA interactions in development and malignancy.

The conservation of mechanisms across development and tumorigenesis and the significant roles of miRNA in both development and tumorigenesis [10], [11], [18]–[22] also motivated our investigation of miRNA–mRNA interactions in both tumors and their cognate developing tissue. Therefore, we intersected the coherent and non-coherent target gene sets observed during cerebellar development with the up- or down-regulated gene sets observed in Ptch+/− medulloblastoma (MB) and compared the logarithmic fold change of expression in tumor with that of the up- or down-regulated non-target genes. As a tissue-specificity control, functional gene sets in murine lung development and lung cancers were studied in parallel. The design of this tissue-specific and temporal-specific functional study of miRNA–mRNA target interaction across development and tumorigenesis is illustrated in Figure 1.

## Results

### The number of miRNA non-coherent targets is equivalent to that of miRNA coherent targets in developing cerebellum and lung tissue

We focused on postnatal days 7 (P7) and P60 for cerebellar development, because the highest level of granule neuron precursor proliferation and migration occurs during P7 and the development of mouse Ptch+/− MB is most closely associated with stage P7 [20], whereas P60 is an adult stage during which miRNA levels are assumed to be stable. Using customized RAKE miRNA microchips [23], we profiled wild-type mouse miRNA expression in developing cerebellum at postnatal stages P7 and P60 (Table 1 and Figure S1). In parallel, we studied the miRNA expression in developing lung at stages P1 and P14, as described in Williams et al. [24]. We have previously reported on total RNA expression in developing mouse cerebellum for P1, P3, P5, P7, P10, P15, P21, P30, P50, and P60 based on the Affymetrix Mu11K arrays [25]. A complete time series of mRNA expression, (also Mu11K arrays), of perfused whole wild-type mouse lung for embryonic days 12, 14, 16, and 18, and postnatal days P1, P4, P7, P10, P14, and P21, covering the five main stages of mouse lung development [26] was also available [27].

**Table 1:** miRNA expression data of developing mouse cerebellum.

miRNA Name	pval (Day 7B vs Day 60C) ranked data	Data P7	Data P60	Log2 FC	Num of coherent gns (Seri. A) using TargetScanS	Num of Non-coherent gns (Seri. A) using TargetScanS	Num of coherent gns (Seri. A) using PITA	Num of Non-coherent gns (Seri. A) using PITA	Num of coherent gns (Seri. A) using picTar	Num of Non-coherent gns (Seri. A) using picTar
**mmu-let-7a**	0.89827	65423	65481	0.0012784	110	99	97	87	109	95
**mmu-mir-124a**	0.0021645	22091	65482	1.5676397	274	249	201	165	163	127
**mmu-mir-125a**	0.064935	8299	56964	2.779041	87	85	141	132	88	78
**mmu-mir-103-1,2**	0.39394	4940	15020	1.6043019	68	84	77	80	113	154
**mmu-mir-9**	0.004329	1354	3855	1.5095031	162	177	153	125	139	157
**mmu-mir-23b**	0.0021645	1262	5891	2.2228006	123	126	161	131	78	82
**mmu-mir-206**	0.0021645	1083	3019	1.4790375	121	116	93	90	111	104
**mmu-mir-15**	0.0021645	680	1402	1.0438797	122	151	164	191	120	152
**mmu-mir-30b**	0.0021645	590	21826	5.209189	191	162	188	164	122	100
**mmu-mir-99b**	0.015152	450	5933	3.7207649	6	6	7	7	5	5
**mmu-mir-221**	0.0021645	426	3083	2.8554096	60	43	61	69	50	49
**mmu-mir-187**	0.39394	399	1002	1.3284219	0	2	11	11	0	3
**mmu-mir-138**	0.0021645	372	854	1.1989334	58	60	71	66	56	50
**mmu-mir-194**	0.0021645	309	4231	3.7753199	55	48	52	54	37	41
**mmu-mir-133**	0.0021645	287	1585	2.4653602	72	69	67	62	66	68
**mmu-mir-21**	0.0021645	275	1555	2.4994111	34	29	59	47	37	26
**mmu-mir-204**	0.0021645	266	4643	4.1255591	70	63	86	91	71	63
**mmu-mir-34a**	0.0021645	155	1873	3.5950108	86	75	86	85	81	77
**mmu-mir-152**	0.0021645	102	313	1.6175935	74	102	109	117	65	92
**mmu-mir-218-1,2**	0.0021645	93	2281	4.6162919	79	111	111	113	77	100
**mmu-mir-182**	0.0021645	86	424	2.3016557	114	115	119	99	130	134
**mmu-mir-146**	0.0021645	85	238	1.4854268	29	22	39	32	23	22
**mmu-mir-7**	0.0021645	38	214	2.4935395	54	54	57	55	53	55
**mmu-mir-101**	0.0021645	34	70	1.0418202	53	55	89	90	94	104
**mmu-mir-139**	0.0021645	33	51	0.6280312	58	54	78	104	54	49
**mmu-mir-223**	0.0021645	32	98	1.6147098	45	34	42	51	35	33
**mmu-mir-137**	0.0021645	24	34	0.5025003	85	80	105	121	72	67
**mmu-mir-96**	0.0021645	23	50	1.1202942	64	55	87	70	133	138
**mmu-mir-128**	0.0021645	7840	53416	2.7683464	98	96	140	126	114	120
**mmu-mir-26a**	0.0021645	4037	65474	4.0195666	134	89	113	81	101	69
**mmu-mir-22**	0.0021645	1411	11129	2.9795341	60	68	74	72	64	65
**mmu-mir-145**	0.0021645	730	2578	1.8202839	93	65	87	86	51	41
**mmu-mir-143**	0.0021645	217	1033	2.2510733	51	40	65	38	45	38
**mmu-mir-27b**	0.0021645	125	907	2.8591745	103	100	152	157	136	144
**mmu-mir-192**	0.0021645	86	579	2.7511548	18	19	18	27	21	17
**mmu-mir-140**	0.0021645	39	41	0.0721498	38	33	61	57	42	42
**mmu-mir-216**	0.0021645	1135	24	−5.5635141	27	23	68	68	23	20
**mmu-mir-375**	0.1	36	8	−2.169925	42	45	7	10	24	27
**mmu-mir-144**	0.93723	24	9	−1.4150375	21	21	112	122	98	108
**mmu-mir-181a**	0.0021645	32848	27769	−0.2423303	125	130	137	168	86	93
**mmu-mir-93**	0.0021645	15075	1561	−3.2716156	72	71	159	151	136	129
**mmu-mir-130**	0.0021645	12033	3819	−1.6557295	99	73	135	103	120	87
**mmu-mir-92-1,2**	0.0021645	11935	364	−5.0351163	128	102	97	86	92	62
**mmu-mir-106**	0.0021645	3563	234	−3.928512	156	120	159	146	135	107
**mmu-mir-217**	0.0021645	450	18	−4.6438562	28	44	69	74	31	37
**mmu-mir-122a**	0.0021645	270	49	−2.4621058	33	27	26	30	30	25
**mmu-mir-155**	0.0021645	214	54	−1.9865795	37	59	56	64	33	47
**mmu-mir-184**	0.041126	88	64	−0.4594316	5	5	6	6	5	5
**mmu-mir-199a-1**	0.17965	86	7	−3.6189098	56	54	128	143	36	51
**mmu-mir-19a**	0.0021645	56	10	−2.4854268	125	124	154	142	131	120
**mmu-mir-33**	0.39394	45	23	−0.9682911	52	34	57	55	43	33
**mmu-mir-142-s**	0.13203	44	8	−2.4594316	40	37	41	58	40	33
**mmu-mir-219**	0.24026	40	23	−0.7983661	43	45	36	31	34	38
**mmu-mir-153**	0.17965	29	19	−0.6100535	75	81	68	89	72	74

pval — the Wilcoxon ranksum test result comparing P7 and P60 ranked sorted miRNA expression;

Log2FC — the logarithmic fold change between P60 and P7 miRNA expression.

TargetScanS [28] computational prediction of targets for 54 conserved miRNAs in developing cerebellum and 59 miRNAs in developing lung was performed. For each miRNA, we identified the coherent target and non-coherent target sets using the P7 and P60 data points in developing cerebellum and likewise we did the test using the P1 and P14 data points in developing lung. Positive correlation between miRNA and mRNA target is considered non-coherent and accordingly negative correlation is considered coherent. In both developing cerebellum and lung, the number of non-coherent targets was equivalent to that of coherent targets for each miRNA, regardless of its level of expression (Table 1 and Table S1). The mean number of coherent targets per miRNA was 76 in developing cerebellum and 66 in developing lung, and the number of non-coherent targets was 72 and 69, respectively.

We performed the same procedure using PITA[29] and picTar[30] target predictions and found similar phenomenon in each case, (the right two columns of Table 1 and Table S1). Likewise, in the following findings we conducted the tests with PITA and picTar predictions as well in addition to TargetScans in order to exclude algorithm-specific artifact. Results of the comparisons are demonstrated in each place where such a purpose is addressed.

### miRNAs can be classified according to the coherence and non-coherence of their target sets in developing cerebellum

**Figure 2:**
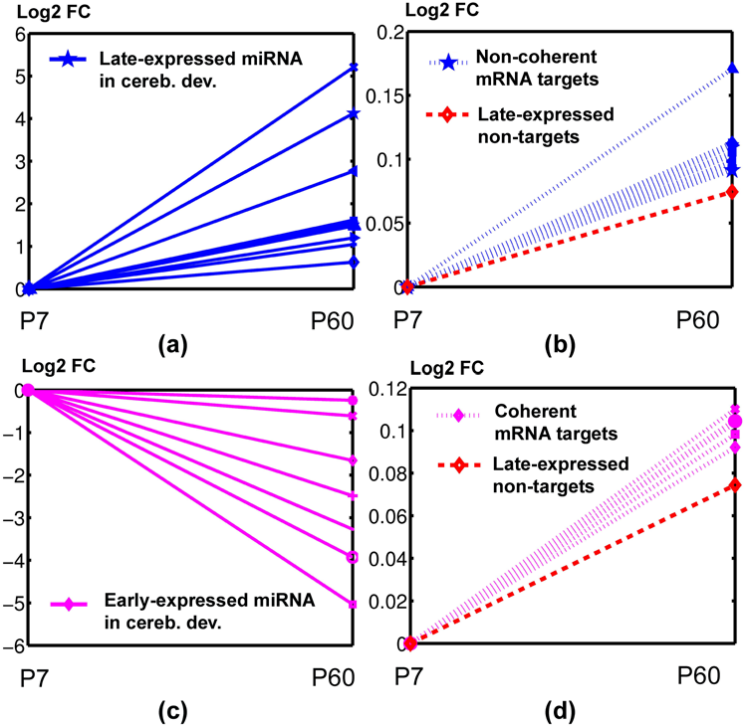
Significant opposite effects of the miRNAs on the coherent and non-coherent target genes in developing cerebellum. (A) Late expressed miRNAs in Table 2. (B) Non-coherent mRNA targets of late expressed miRNAs. (C) Early expressed miRNAs in Table 2. (D) Coherent mRNA targets of early expressed miRNAs. Dashed line represents average of the non-target genes that expressed late in developing cerebellum.

We next examined whether miRNAs can be classified according to the coherence and non-coherence of their targets during development given that we found non-coherent targets to be as common as coherent ones. Using non-target genes as a negative control, we compared the changes in mRNA expression of coherent targets or non-coherent targets for each miRNA with those of changes in the expression of non-target genes that had a positively correlated developmental profile to the target set in test. We then computed the statistic of the tests to identify which miRNAs are significant when their coherent targets are compared with the non-target control set and which are significant when tested for their non-coherent targets. In conjunction with the two types of miRNAs (developmentally early expressed/early-expressed miRNAs and developmentally late expressed/late-expressed miRNAs), there shall be four types of test in all. The tests revealed two significant (Wilcoxon ranksum test p<0.05) miRNA expression patterns during development as demonstrated in Figure 2. The average logarithmic relative expression of miRNA targets at day P60 compared to that of P7 was plotted against that of the corresponding background non-target genes. We use “LNCoh” to denote late-expressed miRNAs significant for their non-coherent targets (Figure 2A shows for the miRNAs, Figure 2B for the corresponding mRNA non-coherent targets), and “ECoh” to denote early-expressed miRNAs significant for their coherent targets (Figure 2C for the miRs, Figure 2D for the corresponding mRNA coherent targets).

**Table 2:** Significant miRNAs in mouse cerebellum development.

Significant miRNAs in cerebellum dev. for their *non-coherent* targets						
miRNA Name	Dev Status	Num and % of non-coherent genes	Log2 (P60/P7) of the miR	Ave. Log FC offset	P-val (a)	P-val (b)
**hsa-mir-15**	**Late**	151/55.31%	**1.044**	**0.029**	**0.0001**	**7.03E-05**
**mmu-mir-124a**	**Late**	249/47.61%	**1.568**	**0.018**	**0.0017**	**0.0010**
**mmu-mir-152**	**Late**	102/57.95%	**1.618**	**0.031**	**0.0020**	**0.0005**
**hsa-mir-9**	**Late**	177/52.21	**1.510**	**0.022**	**0.0020**	**0.0002**
**mmu-mir-30b**	**Late**	162/45.89%	**5.209**	**0.019**	**0.0028**	**0.0099**
**hsa-mir-103-1,2**	**Late**	84/55.26%	**1.604**	**0.031**	**0.0030**	**0.0002**
**hsa-mir-139**	**Late**	54/48.21%	**0.628**	**0.039**	**0.0045**	**0.0004**
**mmu-mir-146**	**Late**	22/43.14%	**1.485**	**0.096**	**0.0063**	**0.0465**
**mmu-mir-206**	**Late**	116/48.95%	**1.479**	**0.035**	**0.0121**	**0.0015**
**mmu-mir-138**	**Late**	60/50.85%	**1.199**	**0.036**	**0.0174**	**0.0108**
**mmu-mir-128**	**Late**	96/49.48%	**2.768**	**0.024**	**0.0218**	**0.0161**
**mmu-mir-204**	**Late**	63/47.37%	**4.126**	**0.017**	**0.0296**	**0.0211**

Significant miRNAs in cerebellum dev. for their *coherent* targets						
miRNA Name	Dev Status	Num and % of coherent genes	Log2 (P60/P7) of the miR	Ave. Log FC offset	P-val (a)	P-val (b)
**hsa-mir-19a**	**Early**	125/50.20%	**−2.485**	**0.031**	**1.5023E-05**	**2.19E-06**
**hsa-mir-106**	**Early**	156/56.52%	**−3.929**	**0.030**	**2.3594E-05**	**3.10E-07**
**hsa-mir-181a**	**Early**	125/49.02%	**−0.242**	**0.029**	**0.0001**	**3.42E-06**
**miR-93**	**Early**	72/50.35%	**−3.272**	**0.030**	**0.0019**	**1.78E-05**
**mmu-mir-153**	**Early**	75/48.08%	**−0.610**	**0.036**	**0.0108**	**0.0088**
**hsa-mir-92-1,2**	**Early**	128/55.65%	**−5.035**	**0.024**	**0.0109**	**0.0001**
**miR-130**	**Early**	99/57.56%	**−1.656**	**0.018**	**0.0111**	**0.0181**

Ave. LogFC Val offset — average offset of the logarithmic fold change (P60/P7 in dev.) calculated for the involved miRNA non-coherent/coherent targets from that of non-miRNA target genes;

P-val (a) —the p-vals calculated for the involved miRNA using the logFC of non-coherent targets/coherent targets vs. non-miRNA-target gene background;

P-val (b) (the p-vals calculated from duplicate dev data).

The graphs in Figure 2 illustrate the opposite effects of the miRNAs on the coherent and non-coherent genes. In the developing cerebellum, 12 of the 36 late-expressed miRNAs were LNCoh and 7 of the 18 early-expressed miRNAs were ECoh (Table 2). Using the prediction by PITA and picTar, we identified a similar set of significant miRNAs. In the case of using PITA prediction, 22 of 36 late-expressed miRNAs were LNCoh-type while 10 of the 18 early-expressed miRNAs wer ECoh-type (Table S2). With picTar prediction, 13 of the late miRNAs were LNCoh-type and 9 of the 18 early miRNAs were ECoh-type (Table S3). Both non-coherent targets for early expressed miRNAs and coherent targets for late expressed miRNAs are not statistically significant compared with the corresponding non-target background gene set. It is noteworthy that as the miRNA expression decreases, the upregulation of coherent targets of the ECoh-type miRNAs is significantly greater than that of the non-target genes and, more surprisingly, the non-coherent targets of the LNCoh-type miRNAs escape even further from miRNA suppression than non-target genes.

Interestingly, both miR-124 (a highly brain-specific miRNA) and miR-9 (a highly functional miRNA in brain development) are expressed late in development and are significant when their non-coherent targets are compared with the non-target control gene set. In comparison, there are far fewer significant miRNAs either for the non-coherence or for the coherence of their targets in the developing lung, where there is no apparent bias towards a particular category (Table S4). These results suggest that many late-expressed miRNAs mediate target non-coherence in a tissue-specific and functional manner.

### Non-coherent target sets of late-expressed miRNAs correspond significantly with processes involving cell-communication among which s*ynaptic transmission* and others co-express multiple miRNAs at a significantly higher level than do non-targets

Based on the finding that late miRNAs are characterized by the non-coherence of their targets, we examined the ontological correlates of the target sets. Among the non-coherent targets of the late-expressed miRNAs, GO terms such as *cell-communication, signal transducer activity, cell differentiation*, and *morphogenesis* were enriched with the non-target background as control. Table 3 summarizes the enriched GO terms of miRNA coherent/non-coherent targets in developing cerebellum. We further investigated whether the non-coherent targets associated with these terms were still significantly enriched against the non-targets associated with the same terms that positively correlate with the non-coherent targets and found *cell-communication* and *cell differentiation* were again significant (Table S5). The test statistic is the logarithmic fold-change of the expression in developing cerebellum as in previous tests. This finding is important in that although the miRNA binding sites for mRNA genes of these GO terms are enriched on a genome scale [16], these functional processes are non-coherent to miRNA suppression.

**Table 3:** The enriched Gene Ontological terms composed of miRNA non-coherent/coherent targets in cerebellum development.

		Gene Ontological terms	p-val	LogFC Val offset	miRNAs										
Non-coherent terms	sort by multiplicity of miRs	‘transmission of nerve impulse’	0.0046	0.1596	mir-128	mir-137	mir-218	mir-27b	mir-143	mir-133	mir-206	mir-152	let-7a	mir-9	mir-138
		‘synaptic transmission’	0.0046	0.1596	mir-128	mir-137	mir-218	mir-27b	mir-143	mir-133	mir-206	mir-152	let-7a	mir-9	mir-138
		‘transport’	0.0028	0.0852	mir-103	mir-128	mir-218	mir-23b	mir-101	mir-21	mir-15	mir-138			
		‘localization’	0.0017	0.0749	mir-103	mir-128	mir-139	mir-218	mir-23b	mir-21	mir-15	mir-138			
		‘transporter activity’	0.0014	0.0941	mir-103	mir-128	mir-218	mir-221	mir-23b	mir-30b	mir-15	mir-138			
	sort by p-vals	‘cell communication’	3.79E-05	0.0759	mir-138										
		‘nucleus’	0.0001	0.0337	mir-9										
		‘membrane-bound organelle’	0.0002	0.0325	mir-9										
		‘cellular process’	0.0001	0.0337	mir-15										
		‘intracellular membrane-bound organelle’	0.0002	0.0324	mir-9										
	sort by offset from non-targets	‘synapse’	0.0047	0.3267	mir-146	mir-34a	mir-206								
		‘metal ion-binding site:Calcium 2’	0.0048	0.2882	mir-128	mir-34a	mir-152	let-7a							
		‘lipid binding’	0.0033	0.2982	mir-34a										
		‘metal ion-binding site:Calcium 1’	0.0048	0.2882	mir-128	mir-34a	mir-152	let-7a							
		‘metal ion-binding site:Calcium 1 (via carbonyl oxygen)’	0.0052	0.2882	mir-128	mir-34a									
Coherent terms	sort by multiplicity of miRs	‘physiological process’	0.0047	0.0280	mir-153	mir-181a	mir-19a	mir-93	mir-142s	mir-92	mir-106				
		‘DNA metabolism’	0.0261	-0.0633	mir-194	mir-23b	mir-145	mir-21	let-7a	mir-138					
		‘cellular physiological process’	0.0033	0.0304	mir-153	mir-181a	mir-19a	mir-93	mir-92	mir-106					
		‘cellular process’	0.0019	0.0297	mir-153	mir-181a	mir-19a	mir-93	mir-92	mir-106					
		‘metabolism’	0.0049	0.0242	mir-181a	mir-19a	mir-93	mir-142s	mir-106						
	sort by p-vals	‘cell’	0.0008	0.0318	mir-153	mir-181a	mir-19a	mir-106							
		‘extracellular matrix structural constituent’	0.0012	-0.1722	let-7a										
		‘cellular process’	0.0019	0.0297	mir-153	mir-181a	mir-19a	mir-93	mir-92	mir-106					
		‘HSA04512:ECM-RECEPTOR INTERACTION’	0.0024	-0.1612	let-7a										
		‘binding’	0.0032	0.0297	mir-181a	mir-19a	mir-93	mir-106							
	sort by offset from non-targets	‘nuclear membrane’	0.0225	-0.2573	mir-23b										
		‘HSA04110:CELL CYCLE’	0.0212	-0.2572	mir-124a										
		‘HSA01430:CELL COMMUNICATION’	0.0135	-0.2084	let-7a	mir-124a									
		‘coiled coil’	0.0125	-0.1946	mir-101										
		‘trimer’	0.0154	-0.1917	let-7a										

p-val — median of the p-vals calculated for the involved miRNAs using the logFC of non-coherent targets/coherent targets vs. non-miRNA-target gene background;

LogFC Val offset — median of the offset of the logarithmic fold change (P60/P7 in dev.) calculated for the involved miRNAs non-coherent/coherent targets from that of non-miRNA target genes;

# of miRNAs — number of associated miRNA incidences in Dev. (common for two duplicates) with the GO terms.

To determine the extent to which the non-coherent targets for each miRNA in terms of GO terms differ from the non-target genes in developing cerebellum, we investigated the average logarithmic fold-change of mRNA expression from P7 to P60 and compared the result with the value of the corresponding non-target genes that had a positive-correlated developmental profile to the target set. Many enriched GO terms were co-expressed with the late-expressed miRNAs at significantly higher levels than that of non-target genes, with an average fold-change difference of 55%.

We sorted the above obtained GO terms based on their non-coherent targets' offset from non-target background genes in terms of logarithmic fold-change from P7 to P60, their statistical significance in the enrichment test, and their multiplicity of miRNAs, respectively (Table 3). Among the non-coherent ontological gene sets, the terms *Metal ion-binding site:Calcium* and *Synaptic transmission* ranked at the top if the three ranks were weighted equally. Having the most number of putative binding miRNAs, *Synaptic transmission* exhibited a 140% greater fold-change from P7 to P60 compared with the average non-target late-expressed genes (p<0.009). A total of 11 miRNAs, let-7, miR-9, miR-206, miR-138, miR-133, miR-152, miR-137, miR-128, miR-143, miR-27b and miR-218 were co-expressed by 18 synaptic transmission target genes (Table S6).

In order to understand the robustness of the non-coherence of the afore identified pathways in the dynamics of cerebellum development, we computed the differential expression of target genes in the intermediate time points (P10, P15, P21, P30) relative to P7 respectively, versus the miRNA differential expression at P60 relative to P7. A similar list of pathways were found to be significant in developing cerebellum at these 4 stages (Table S7). Moreover, the statistical significances at later stages P21 and P30 are higher than at early stages P10 and P15, which demonstrate progressive nature of developmental non-coherence of mRNA target of late miRNAs.

Synaptic transmission is the essential process of transferring signals between neurons in the CNS [31]. Functioning mainly in chemical synapses, the 18 synaptic transmission genes cover the different stages of both presynaptic and postsynaptic neurotransmission at the synapse. For example, SYT1 and SNAP-25 are presynaptic proteins involved in neurotransmitter release, whereas GABARAPL1 is a postsynaptic receptor. Some synaptic transmission genes, such as RIT2, are exclusively expressed in neurons. We examined whether there is a hierarchical relationship among the enriched GO terms and their relation, if any, to *synaptic transmission*. We identified two pedigree sub-trees of GO terms that were closely related in the context of the synapse: a *cell communication*-rooted tree branching to *synaptic transmission* and a *localization*-rooted tree branching to *exocytosis,* which is the process that releases neurotransmitters into the synaptic cleft.

We further investigated whether the non-coherent target set of *synaptic transmission* was significant using the non-target *synaptic transmission* genes as controls because on average, late-expressed synaptic transmission non-target genes have a higher fold-change from P7 to P60 than do other non-target genes. Again, the non-coherent *synaptic transmission* genes were significant in this case for each miRNA involved (p<0.05). Moreover, the processes in the two sub-trees of GO terms (mentioned above) are generally among the most significant. Comparison of the non-coherent *exocytosis* targets with the non-target *exocytosis* genes revealed a similar phenomenon. This finding suggests the enriched non-coherent GO processes are not isolated events, but rather functionally consistent phenomena mediated by miRNA.

We performed the same statistical test and analysis for the enrichment of non-coherent GO processes using the targets predicted by PITA and picTar. Comparing the results (Table S8, S9) with the findings using TargetScanS prediction, we found that Synaptic transmission again ranked at the top and the related GO processes are included in the list of significant terms. The coherent ontological gene sets are also tested (Table S8, S9) and we found discrepancy in results using different predictors. In particular, the most enriched coherent terms from TargetScanS include basic processes such as Physiological process, cellular process, DNA metabolism and chromatin assembly/disassembly that are not largely represented in PITA and picTar target predictions and thus are not identified as significant ones using the other two predictors.

### A common miRNA–mRNA co-expression program of non-coherent target sets of GO processes is shared between developing cerebellum and medulloblastoma (MB): example of two sub-trees of GO terms

The functional enrichment of groups of non-coherent ontological target sets reveals a positive output of targets toward the corresponding miRNAs in developing cerebellum. We examined whether mRNA targets avoid miRNA suppression in malignant brain tumors. We identified the intersecting sets of coherent/non-coherent targets in developing cerebellum and the up/down targets in mouse Ptch+/− MB and tested them against the up/down non-target background genes for enriched GO terms (Table S10). All significant non-coherent ontological target sets for late miRNAs were downregulated and all significant coherent ontological target sets for late miRNAs were upregulated in MB.

**Figure 3:**
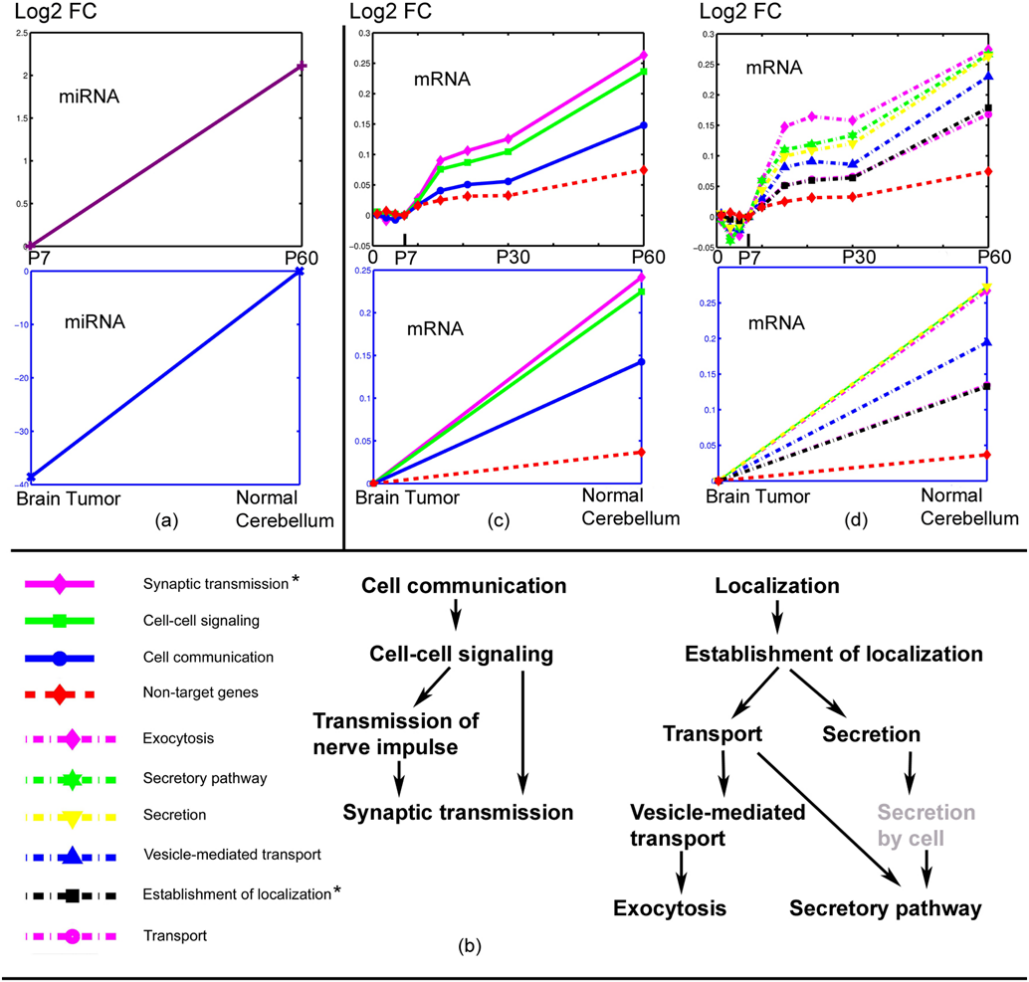
Common miRNA–mRNA co-expression pattern. Shared non-coherent ontological gene sets between brain development and tumors (A) average miRNA profiles in developing cerebellum and tumor, (B) legend and the two sub-tree hierarchy of the synaptic transmission-related processes. (C,D) developmental mRNA profiles of the brain-specific neurologic terms that significantly avoid miRNA suppression. * *Synaptic transmission* and *Transmission of nerve impulse* share the same set of mRNA target genes; *Establishment of localization* and *Localization* share the same set of mRNA target genes.

As in developing cerebellum, the groups of non-coherent GO processes for late-expressed miRNAs, including *synaptic transmission*, were significantly different from non-target downregulated mRNA in MB (Table 4). Again, the GO processes were composed of two sub-trees (Figure 3B), as in development, for shared miRNAs, such as miR-9, miR-206, miR-138, miR-133, miR-152, and miR-128. Given that Ptch+/− MB is most closely associated with stage P7 [20] in developing cerebellum, we compared the adult normal samples to the Ptch+/− MB and plotted the average logarithmic fold-change of mRNA expression of adult normal tissue over Ptch+/− MB. Figure 3C shows the cell communication-rooted sub-tree branching to the *synaptic transmission* logarithmic fold-change in both developing tissue and tumor, and Figure 3D shows the fold-change profiles of the localization-rooted sub-tree branching to *exocytosis*. In both figures, the corresponding non-target genes were used as controls. Interestingly, not only the two sub-trees of GO terms were shared, the magnitudes and orders of the terms in MB and developing cerebellum were similar. As before, we tested the non-coherent *synaptic transmission* target sets against non-target down-regulated *synaptic transmission* genes in MB and found the non-coherent miRNA targets were still significant, as were the other GO terms in the two shared sub-trees. The two sub-trees of GO processes are also shared between developing cerebellum and Ptch+/− MB when picTar and PITA predictions are used (Table S11, S12). For synaptic transmission, miR-128, miR-27b, miR-133, miR-206, miR-152 and miR-9 are shared between development and tumor using picTar prediction; miR-128, miR-140, miR-27b, miR-22, miR-133, miR-223 and miR-152 are shared using PITA prediction.

**Table 4:** The statistic significance and other quantifications of the shared miRNA non-coherent GO terms shared between brain tumor and cerebellum development.

GO terms	p-val in Dev.	LogFC Val offset in Dev.	# of miRs in Dev.	p-val in MB	LogFC Val offset in MB	# of miRs in MB	# of common genes	significant miRNAs shared btw Ptch+/− MB and development						
**‘transmission of nerve impulse’**	0.0046	0.1596	11	0.0043	−0.1944	8	18	**mir-128**	**mir-218**	**mir-133**	**mir-206**	**mir-152**	**mir-9**	**mir-138**
**‘synaptic transmission’**	0.0046	0.1596	11	0.0043	−0.1944	8	18	**mir-128**	**mir-218**	**mir-133**	**mir-206**	**mir-152**	**mir-9**	**mir-138**
**‘cell communication’**	0.0000	0.0759	1	0.0029	−0.0945	4	19	**mir-138**						
**‘transport’**	0.0028	0.0852	8	0.0008	−0.1051	5	21	**mir-128**	**mir-218**	**mir-138**				
**‘cell–cell signaling’**	0.0039	0.1265	6	0.0015	−0.1964	4	8	**mir-128**	**mir-133**					
**‘localization’**	0.0017	0.0749	8	0.0004	−0.1046	4	30	**mir-128**	**mir-218**					
**‘establishment of localization’**	0.0017	0.0749	8	0.0004	−0.1046	4	30	**mir-128**	**mir-218**					
**‘secretion’**	0.0080	0.1748	3	0.0081	−0.1637	2	6	**mir-103**	**mir-128**					
**‘exocytosis’**	0.0018	0.2142	1	0.0010	−0.2354	1	4	**mir-128**						
**‘vesicle-mediated transport’**	0.0054	0.1456	2	0.0091	−0.1637	2	7	**mir-103**	**mir-128**					
**‘secretory pathway’**	0.0095	0.1825	2	0.0010	−0.2354	1	4	**mir-128**						

p-val — median of the p-vals calculated for the involved miRNAs using the logFC of non-coherent targets targets vs. non-miRNA-target gene background;

LogFC Val offset — offset of the median of the logarithmic fold change (P60/P7 in dev.) calculated for the involved miRNAs non-coherent targets from that of non-miRNA target genes;

# of miRNAs — number of associated miRNA incidences (common for two duplicates) with the GO terms;

# of common genes — number of common miRNA non-coherent targets shared by developing cerebellum tissue and MB tumor for the associated term.

We then examined the miRNA expression in brain cancers. We obtained CNS cancer cell line miRNAs from the NCI-60 database [32], which were histologically glioblastoma. Glioblastoma is a primary CNS tumor that sometimes occurs in the cerebellum. Compared with normal P60 cerebellum, almost all the late miRNAs in developing cerebellum were downregulated in these CNS tumor cell lines (Figure 3A). All except one of the involved miRNAs for the shared two sub-trees of GO terms were downregulated, and the expression of that one was not changed (Table S13).

In addition, we tested the human MB cell line and found similar sharing of significant non-coherent ontological target sets, including *synaptic transmission* and *exocytosis,* between MB and developing cerebellum (Table S10). Tests in developing lung and lung cancers performed in parallel revealed that no significant non-coherent ontological gene sets were shared between them.

Together, these findings indicate that there is common program of process-specific miRNA–mRNA co-expression between developing cerebellum and CNS tumors. In particular, the brain-specific neurologic process *synaptic transmission*, and two closely related processes, *vesicle-mediated transport* and *exocytosis,* significantly avoid regulation by the gain of function of multiple miRNAs in developing cerebellum as well as by the same miRNA's loss of function in brain tumors.

### miRNA–mRNA co-expression in brain development and malignancy are tissue-specific

In addition to the fact that fewer miRNAs were found significant for their target's coherence or non-coherence in developing lung than in developing cerebellum (Table S4), there were also very few common significant GO terms in each of the types defined as either early or late and coherent or non-coherent (Table S14).

Between developing cerebellum and lung, only two generic GO terms are common including *cellular physiological process and binding* (Table S14), while overall there were 164 significant non-coherent ontological gene sets in the cerebellum. Both these two categories are significantly non-coherent to miR-15. Although *synaptic transmission* target set was also significantly non-coherent in developing lung, it involved only miR-140 and miR-200b, which were different miRNAs from those in developing cerebellum. In developing lung, no group of GO terms was significantly associated with *synaptic transmission*, in contrast to developing cerebellum. Regulation of the *actin cytoskeleton* and *MAPK signaling pathway* are among the identified lung development-specific non-coherent ontological target sets for miR-140, which is significant in lung development for its target non-coherence (Table S4).

Far fewer significant miRNAs were found in developing lung than in developing cerebellum with picTar and PITA predictions (Table S15). There is one significant miRNA for its non-coherent targets (miR-146) and one for coherent targets (miR-204) in the case of picTar while there are no significant miRNAs when PITA is used. Comparing the enrichment of GO processes between developing lung and cerebellum, we found *Metal ion transport* and *MAPKKK cascade* are commonly significantly non-coherent to miR-15 and that *Phosphorylation* is commonly significantly coherent to miR-181 using picTar prediction (Table S16). There are no significant commonly enriched GO processes found when PITA prediction is used.

Unlike the shared program described between developing cerebellum and MB, only three terms such as *activator*, *DNA binding*, and *DNA metabolism*, were shared between small cell lung cancer upregulated genes and coherent targets in developing lung, involving miR-30, miR-200a, and miR-9, respectively. We did not find any shared processes between small cell lung cancer upregulated genes and coherent targets in developing lung using picTar or PITA prediction.

## Discussion

This study focused on co-expressed miRNA-target pairs in temporally-specific and tissue-specific mammalian CNS development and malignancy. Many of the late-expressed miRNAs in developing cerebellum were characterized by their target non-coherence. Further identification of the shared CNS-specific network of enriched co-expressed GO terms surrounding *synaptic transmission* between cerebellar development and brain tumors confirmed the tissue and process specific mRNA co-expression with multiple miRNAs.

It is difficult to explain these findings based only on the mutual exclusion of miRNAs and targets. Although cell-type variety may facilitate the mutual exclusion, here the miRNA targets were compared with non-target genes that had a positively-correlated developmental profile to the target set using the same assay with the same averaging of cell-types, thus minimizing the effects of cell-type. In addition, the limited number of cell types in the cerebellum and the prevalence of some of the significant miRNAs in the CNS [33] make it more difficult to apply the mutual exclusion model. Furthermore, the identified *synaptic transmission* process is hard to explain as specific to a particular neuron.

Transcription factors and miRNA interactions might contribute to the phenomenon of miRNA–mRNA co-expression. Feedback loops between these two types of transcription regulators have been extensively reported [1], [12], [34]–[37]. A recent computational model by Shalgi et al. suggests that in a significant fraction of such interactions transcription factors regulate the miRNA or are regulated by miRNA and these forms of feed-forward loops are often observed in developmental processes. Consistent with the abundant sites in neuronal tissues of highly expressed genes [15], Tsang et al. reported co-expression of miRNA-target pairs in neuronal tissue computed by a score based on the number of conserved binding sites [38]. Among the two promoter-miRNA-target interaction models described by Tsang et al.[38], a circuit named Type I, which is equivalent to the special case of a feed-forward loop described by Shalgi et al.[36], recurs in different tissues and might explain the co-expression. Among the brain-enriched miRNAs, however, only miR-7 and miR-103 are consistently reported to be involved in the Type I circuit. For brain tissues, miR-9 and miR-128b are Type I, although miR-128b is not found in the motor neuron data [38]. As miR-9 is reported to have a matched binding motif with neuronal repressor NRSF/REST [39], NRSF might be a promoter that acts in the Type I circuit. Interestingly, recent findings of the in vivo binding partners of NRSF show *synaptic transmission* and other closely related GO terms among the most significant [40]. When compared with the 18 synaptic transmission genes evaluated in this study, however, only 5 genes (GAD1, CACNA1E, NPTX1, DLG4, and GAD2) are among those on the NRSF list. *Exocytosis* genes are not among the list of NRSF binding partners. In addition, the fact that NRSF is not significantly differentiated in brain tumors suggests that NRSF might not form a Type I circuit with miR-9 in brain tumors.

Small dsRNAs can induce transcription activation [41]–[42], which provides another perspective of the mRNA co-expression with miRNA–miRNA-mediated activation. Three genes, E-cadherin, P21, and VEGF, are induced by dsRNAs in the 5′ promoter region in human cancer cell lines [42]. In cerebellar development, VEGF is co-expressed with late-expressed miR-125, whereas E-cadherin and P21 are either not significantly changed or are co-expressed with late miR-9 and miR-22, respectively, in another series (personal communication with J.M. Lee). In addition, data from the RIKEN Brain Science Institute show that E-cadherin is late-expressed in murine cerebellar development. Interestingly, enrichment of miRNA core motifs are reported in the 5′ UTR compared with non-target motifs, and particularly the enrichment of reverse complementary miRNA core motifs in the 5′ UTR appears more frequently in the co-expressed genes of miR-124 than that in 3′ UTR [43], which raises a question as to whether the miRNAs are likely to induce expression from the 5′UTR. A survey of the 5′ UTR patterns of the synaptic transmission genes for 7-nt miRNA motifs shows that the significant miRNAs shared between cerebellar development and MB match various *synaptic transmission* genes. MiR-15 has the greatest degree of multiplicity of 5′ UTR matches with *synaptic transmission* for reverse complementary seed sequences among the significant late miRs. In Xenopus embryonic development, miR-15 regulates Nodal signaling and acts at the crossroads of Nodal signaling and WNT signaling [44]. Intriguingly, miR-15 is found most significant for its targets non-coherence, especially for signal transduction related functions in mouse development (Table S17) while target gene acvr2 is coherent to miR-15 consistent with that in [44].

Recently miRNA-target interactions have been approached in terms of translational repression of the target proteins. Substantial amount of miRNA inhibitions of translation are identified [45]–[46]. Taking into account of this alternative mechanism of miRNA regulation, the miRNA–mRNA co-expression might represent a negative feedback response at the level of translational repression. For example, Baek et al[45] has shown there is a significant cohort of genes were depressed during the protein synthesis with little or no change of mRNA expression although the depression is relatively modest compared with many other targets.

In this manuscript, we attempted to categorize the co-expressed miRNA-target pairs with regard to their functions and temporal-tissue specificity. Although the exact mechanism for the tissue and process specific miRNA–mRNA co-expression observed in the CNS remains to be clarified, our findings point to biologic processes that are likely part of the mechanism of interest. Knowledge of the significant miRNAs and processes shared between cerebellar development and MBs may facilitate target selection for brain tumor therapy.

## Materials and Methods

### miRNA in situ chip data analysis

miRNAs profiled at P7 and P60 of postnatal mouse cerebellum were hybridized on customized RAKE microarray chips with approximately 1700 probes. Significant differences between probes for the same miRNA from P7 to P60 were determined using a Wilcoxon rank sum test and the logarithmic fold-changes in expression were calculated. Fold-changes in relative expression of miRNAs during lung development were obtained from Williams et al.[24].

### Prediction, mRNA data sets, coherent, and non-coherent target sets

TargetScanS [28], PITA[29] and picTar[30] target predictions are obtained from the respectively internet sites. There were 54 conserved miRNAs commonly present in the cerebellar development miRNA data set and there were 59 conserved miRNAs commonly present in the lung development miRNA data set. Mouse development mRNA data sets and MB mRNA microarray data are as described in Kho et al.[20]. Homologous genes were identified between mouse microarray chip probes and the human genome, resulting in 6790 homologous genes in the mouse cerebellar development data series and 6356 homologous genes in the mouse lung development data series. Coherent and non-coherent target sets in each tissue during development were calculated as described previously.

### Significance test of miRNAs

Significance of the change in expression during development for both the coherent target set and the non-coherent target set of each miRNA were assessed using a Wilcoxon rank sum test against the corresponding non-target control set of genes. For example, the logarithmic fold-change of expression from P7 to P60 of the non-coherent target set of a late-expressed miRNA was tested against the late-expressed non-target genes, whereas the coherent target set of an early-expressed miRNA was tested against the early-expressed non-target genes.

### GO (Gene Ontology), other functional terms, and significant GO terms

Gene sets from GO, BBID (Biological Biochemical Image Database), Biocarta, and Kegg pathways were obtained from DAVID Bioinformatics Resource (http://david.abcc.ncifcrf.gov). Each functional set was intersected with the coherent target set and non-coherent target set of each miRNA and significant coherent ontological target sets or non-coherent ontological target sets were identified via a Wilcoxon rank sum test using Matlab (MathWorks; http://www.mathworks.com) against the corresponding non-target genes that had a positive-correlated developmental profile to the target set. In order to correct for multiple testing, we conducted Holms-Bonferroni adjustment according to the smallest p-value for each GO term from the Wilcoxon rank sum test (Table S18). In all three cases with TargetScans, picTar and PITA target predictions, synaptic transmission and related processes appear in the corrected top GO term list.

### Robustness of GO analysis

We tested the enrichment of the non-coherent ontological terms for late miRNAs using *sigPathway* R package in the background of non-target late genes in developing cerebellum. The ontological terms are the above gene sets that intersect with gene sets of developmentally non-coherent late miRNA targets. *sigPathway* is an independent GO pathway analysis package [47]. Synaptic transmission and other related ontological get sets again are found significantly non-coherent to late miRNAs in developing cerebellum. The same test of the ontological enrichment of non-coherent miRNA targets in mouse Ptch+/− MB samples and human MB cell lines using *sigPathway* show a similar list of top pathways (Table S19).

### 5′UTR miR core motif match

The 5′ UTR sequences were obtained from the database developed by Mignone et al.[48]. miRNA core motifs (7nt) were searched for in the 5′ UTR of the genes involved in *synaptic transmission, exocytosis,* and *chromosome* categories (Table S20, S21, S22).

### Validation in cerebellar development duplicate data set

All the significant target sets of miRNAs and GO terms were tested/cross-tested in the two duplicate developmental cerebellum mRNA expression series. There are in all 367 inconsistent genes among 6790 genes in terms of correlation of the expressions and 135 inconsistent among 2633 target genes. There are no inconsistent synaptic transmission genes. The correlations of the gene expressions are included in Table S6. The genes predicted by PITA and picTar for the GO processes in Table S6 are listed in Table S23 and Table S24 respectively.

## Supporting Information

Figure S1 Heat-map image of the logarithmic expression of miRNAs in developing cerebellum P7 and P60.
(1.17 MB TIF)

Table S1 miRNA expression data of developing murine lung.
(0.07 MB PDF)

Table S2 Significant miRNAs in developing cerebellum using PITA prediction.
(0.01 MB PDF)

Table S3 Significant miRNAs in developing cerebellum using picTar prediction.
(0.01 MB PDF)

Table S4 Significant miRNAs developing lung.
(0.01 MB PDF)

Table S5 Enriched non-coherent GO terms for late expressed miRNAs in developing cerebellum compared with non-targets of the same term.
(0.01 MB PDF)

Table S6 Enriched processes of non-coherent genes of miRNA targets common in cerebellum development and Medulloblastoma.
(0.02 MB PDF)

Table S7 Non-coherent gene ontological terms in developing cerebellum at days P10, P15, P21, and P30.
(0.06 MB XLS)

Table S8 Significant GO terms in developing cerebellum comparing targets with non-target control set (PITA target prediction is used).
(0.01 MB PDF)

Table S9 Significant GO terms in developing cerebellum comparing targets with non-target control set (picTar target prediction is used).
(0.01 MB PDF)

Table S10 Summary of the enriched GO terms of late-expressed miRNA coherent/non-coherent targets in murine Ptch+/− MB and human MB cell line.
(0.03 MB PDF)

Table S11 The statistic significance and other qualifications of the shared miRNA non-coherent GO terms between brain tumor and development (picTar target prediction is used).
(0.01 MB PDF)

Table S12 The statistic significance and other qualifications of the shared miRNA non-coherent GO terms between brain tumor and development (PITA target prediction is used).
(0.02 MB XLS)

Table S13 miRNA expressions in cerebellum development and rank change in NCI CNS tumors;
(0.02 MB XLS)

Table S14 Common Enriched GO terms in developing Cerebellum and Lung.
(0.02 MB XLS)

Table S15 Significant miRNAs for their targets' coherence or non-coherence in developing murine lung (picTar prediction is used).
(0.01 MB XLS)

Table S16 Common Enriched GO terms in developing Cerebellum and Lung (picTar prediction is used).
(0.02 MB XLS)

Table S17 Ranksum test of non-coherent targets of miR-15 against non targets in the same GO category.
(0.44 MB XLS)

Table S18 Holm correction for non-coherent Go terms in developing cerebellum (including results from three predictions: TargetScanS, PITA and picTar).
(0.03 MB XLS)

Table S19 List of Top Pathways of non-coherent targets of late miRNAs in developing murine cerebellum, Ptch+/− MB and human MB cell line using sigPathway R package.
(0.04 MB XLS)

Table S20 5′ UTR of Synaptic Transmission Genes that match miRNAs.
(0.15 MB XLS)

Table S21 5′ UTR of Exocytosis Genes that match miRNAs.
(0.06 MB XLS)

Table S22 5′ UTR of Chromosome Genes that match miRNAs.
(0.15 MB XLS)

Table S23 Enriched processes of non-coherent genes of miRNA targets common in cerebellum development and Medulloblastoma (PITA prediction is used).
(0.05 MB XLS)

Table S24 Enriched processes of non-coherent genes of miRNA targets common in cerebellum development and Medulloblastoma (picTar prediction is used).
(0.03 MB XLS)
