# Correction: Tissue and Process Specific microRNA–mRNA Co-Expression in Mammalian Development and Malignancy

**DOI:** 10.1371/annotation/1cdc7975-50d7-40a5-99ca-83580df2982f

**Published:** 2009-05-12

**Authors:** Hongye Liu, Isaac S. Kohane

The second author's name is misspelled. The correct name is Isaac S. Kohane. 

