# Supplementary figures and images for: Tissue and Process Specific microRNA–mRNA Co-Expression in Mammalian Development and Malignancy
Source: PLoS One. 2009 May 5;4(5):e5436. doi: 10.1371/journal.pone.0005436 (PMC2673043; doi:10.1371/journal.pone.0005436)

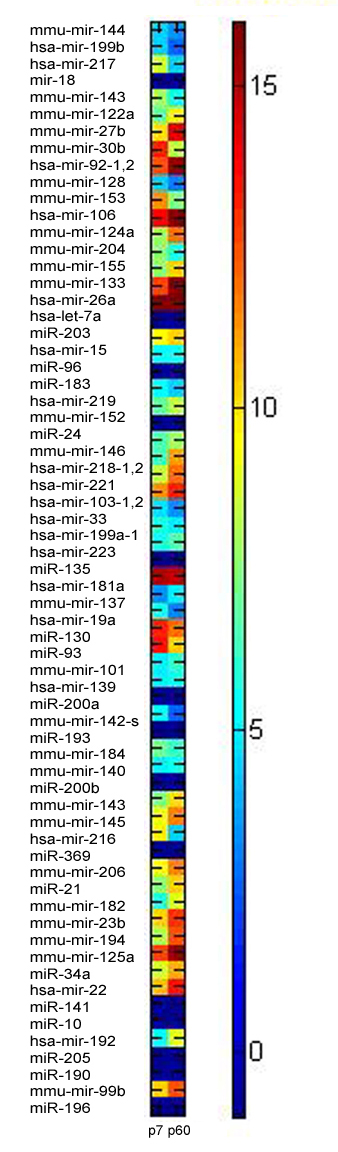

Supplement: Figure S1 — Heat-map image of the logarithmic expression of miRNAs in developing cerebellum P7 and P60. (1.17 MB TIF) [file pone.0005436.s001.tif]
