# Supplementary material for: Tissue and Process Specific microRNA–mRNA Co-Expression in Mammalian Development and Malignancy
Source: PLoS One. 2009 May 5;4(5):e5436. doi: 10.1371/journal.pone.0005436 (PMC2673043; doi:10.1371/journal.pone.0005436)
Supplement: Table S1 — miRNA expression data of developing murine lung. (0.07 MB PDF) [file pone.0005436.s002.pdf]

Supple. Table 1: miRNA expression data of developing mouse lung

miRNA Name	D1 ΔCT	D14 ΔCT	Number of coherent genes using TargetScanS	Number of Non-coherent genes using TargetScanS	Number of coherent genes using PITA	Number of Non-coherent genes using PITA	Number of coherent genes using picTar	Number of Non-coherent genes using picTar
miR-144	18.64973	18.06525	21	18	105	126	96	97
miR-27	10.61053	7.411875	92	94	129	173	116	148
miR-30	9.588775	6.046125	150	190	144	209	83	126
miR-25/32/92/367	8.19715	7.10915	90	120	74	107	63	83
miR-17/20/106	17.75553	16.30388	100	161	107	195	90	141
miR-155	19.01839	18.46817	38	53	52	64	31	43
miR-133	10.3994	7.888725	67	64	54	71	63	62
miR-26	5.258675	3.074125	98	112	83	106	73	84
let-7/miR-98	6.344963	5.98065	98	106	87	102	90	105
miR-15/16/195	10.91528	8.10125	114	143	154	196	117	141
miR-96	13.5754	12.88683	48	64	59	92	96	159
miR-148/152	9.598825	9.29305	69	94	97	128	60	86
miR-146	8.9214	5.821775	24	23	31	37	21	20
miR-221/222	10.64427	9.70605	48	47	50	76	48	48
miR-103/107	7.768275	6.5137	52	93	61	92	113	138
miR-223	9.439363	8.463225	38	36	46	48	35	28
miR-139	13.3194	12.39555	54	53	80	106	48	50
miR-142-3p	8.28365	4.737925	35	39	47	50	31	42
miR-140	8.94415	7.200783	23	41	46	68	29	49
miR-145	6.77855	5.0909	71	80	74	95	37	52
miR-216	23.60846	22.13838	25	21	63	70	27	13
miR-21	7.355375	6.3379	30	31	49	52	28	32
miR-182	11.46359	10.73743	88	129	90	123	100	153
miR-23	8.281625	5.119725	108	132	138	154	72	82
miR-194	12.17366	10.27008	56	41	48	54	41	33
miR-125	6.693525	4.647325	74	92	130	142	67	90
miR-34	9.513425	8.518383	59	93	67	98	56	93

miR-99/100	9.19665	6.454675	5	7	11	5	6	5
miR-187	12.86079	11.0627	1	1	10	11	1	2
miR-9	16.55438	14.92983	150	170	128	145	136	151
miR-138	21.40277	20.72238	58	53	55	79	54	49
miR-122	15.06327	18.35255	30	26	25	30	26	26
miR-128	16.72123	16.916	109	75	139	127	131	95
miR-124	16.95443	17.18138	279	211	216	143	155	117
miR-204/211	13.10046	13.18258	64	60	97	77	63	64
miR-219	16.10836	16.12708	54	33	43	28	44	29
miR-218	8.471388	8.82405	90	86	119	100	84	81
miR-199a*	5.094263	5.40965	59	40	152	112	51	31
miR-181	8.292838	9.457625	135	103	179	130	103	72
miR-137	14.86303	16.33598	94	64	124	99	84	51
miR-130/301	5.569475	5.25505	96	63	141	92	112	82
miR-93/302/372/37[illegible]	21.17381	21.30805	79	56	188	119	153	101
miR-184	15.46129	16.6552	5	4	8	4	5	4

red-- early miRNA **blue-- late miRNA**

ΔCT --- The relative expression of individual miRNA determined by normalization, the lower the value the higher the expression (Williams et al. 06)
