# Supplementary material for: Tissue and Process Specific microRNA–mRNA Co-Expression in Mammalian Development and Malignancy
Source: PLoS One. 2009 May 5;4(5):e5436. doi: 10.1371/journal.pone.0005436 (PMC2673043; doi:10.1371/journal.pone.0005436)
Supplement: Table S2 — Significant miRNAs in developing cerebellum using PITA prediction. (0.01 MB PDF) [file pone.0005436.s003.pdf]

Supple. Table 2: (a) Significant miRNAs in developing cerebellum for their *non-coherent* targets using PITA target prediction

miRNA Name	Dev Status	Num and % of non-coherent genes	Log2 (P60/P7) of the miR	Ave. Log FC offset	P-val (a)	P-val (b)
miR-218	Late	113/50.45%	4.616292	0.036427	2.18E-05	2.71E-06
miR-128	Late	126/47.37%	2.768346	0.038908	0.000314	1.81E-05
miR-9	Late	125/44.96%	1.509503	0.028307	0.000566	0.025436
miR-223	Late	51/54.84%	1.61471	0.060392	0.000798	0.000105
miR-101	Late	90/50.28%	1.04182	0.039592	0.000936	4.95E-05
miR-22	Late	72/49.32%	2.979534	0.032203	0.001139	0.000302
miR-15	Late	191/53.80%	1.04388	0.026339	0.001587	0.005781
miR-152	Late	117/51.77%	1.617594	0.030899	0.001633	0.000511
miR-26a	Late	81/41.75%	4.019567	0.03018	0.001645	6.97E-07
miR-138	Late	66/48.18%	1.198933	0.042794	0.001938	0.000258
miR-140	Late	57/48.31%	0.07215	0.050947	0.002863	0.000792
miR-30b	Late	164/46.59%	5.209189	0.02391	0.003665	6.23E-06
miR-23b	Late	131/44.86%	2.222801	0.028613	0.007018	0.000354
miR-21	Late	47/44.34%	2.499411	0.033742	0.008013	2.51E-05
miR-34a	Late	85/49.71%	3.595011	0.032114	0.00816	0.019762
miR-124a	Late	165/45.08%	1.56764	0.020025	0.01043	0.023291
miR-103-1,2	Late	80/50.96%	1.604302	0.031486	0.011758	9.05E-05
miR-143	Late	38/36.89%	2.251073	0.041221	0.01301	0.006364
miR-137	Late	121/53.54%	0.5025	0.019597	0.020149	0.000397
miR-146	Late	32/45.07%	1.485427	0.052332	0.020629	0.017074
miR-27b	Late	157/50.81%	2.859174	0.021974	0.021523	3.2E-05
miR-204	Late	91/51.41%	4.125559	0.019943	0.04274	0.001557

(b) Significant miRNAs in developing cerebellum for their *coherent* targets using PITA prediction

miRNA Name	Dev Status	Num and % of coherent genes	Log2 (P60/P7) of the miR	Ave. Log FC offset	P-val (a)	P-val (b)
miR-106	Early	159/52.13%	-3.92851	0.032273	4.52E-05	1.19E-07
miR-153	Early	68/43.31%	-0.61005	0.062804	0.000132	9.69E-07
miR-93	Early	159/51.29%	-3.27162	0.026879	0.001084	8.17E-06
miR-181a	Early	137/44.92%	-0.24233	0.026831	0.002137	4.2E-06
miR-33	Early	57/50.89%	-0.96829	0.055088	0.002659	0.004891
miR-144	Early	112/47.86%	-1.41504	0.025118	0.002885	3.22E-07
miR-122a	Early	26/46.43%	-2.46211	0.055649	0.005033	0.001678
miR-130	Early	135/56.72%	-1.65573	0.020435	0.02219	0.010855
miR-19a	Early	154/52.03%	-2.48543	0.015592	0.032569	0.000428
miR-219	Early	36/53.73%	-0.79837	0.033928	0.041059	0.000461
