# Supplementary material for: Tissue and Process Specific microRNA–mRNA Co-Expression in Mammalian Development and Malignancy
Source: PLoS One. 2009 May 5;4(5):e5436. doi: 10.1371/journal.pone.0005436 (PMC2673043; doi:10.1371/journal.pone.0005436)
Supplement: Table S3 — Significant miRNAs in developing cerebellum using picTar prediction. (0.01 MB PDF) [file pone.0005436.s004.pdf]

**Supple. Table 3: (a) Significant miRNAs in developing cerebellum comparing their *non-coherent* targets with non- target control gene set using picTar target prediction**

| miRNA Name | Dev Status | Num and % of non-coherent genes | Log2 (P60/P7) of the miR | Ave. Log FC offset | P-val (a) | P-val (b) |
|---|---|---|---|---|---|---|
| miR-15 | Late | 152/55.88% | 1.04388 | 0.033519 | 2.49E-05 | 6.56E-06 |
| miR-103 | Late | 154/57.68% | 1.604302 | 0.032488 | 5.38E-05 | 2.91E-06 |
| miR-133 | Late | 68/50.75% | 2.46536 | 0.045648 | 0.001881 | 0.048472 |
| miR-139 | Late | 49/47.57% | 0.628031 | 0.04054 | 0.005143 | 0.003694 |
| miR-34a | Late | 77/48.73% | 3.595011 | 0.037374 | 0.006268 | 0.0473 |
| miR-204 | Late | 63/47.01% | 4.125559 | 0.021657 | 0.007438 | 0.025169 |
| miR-128 | Late | 120/51.28% | 2.768346 | 0.023364 | 0.010097 | 0.005491 |
| miR-152 | Late | 92/58.60% | 1.617594 | 0.02482 | 0.01207 | 0.002477 |
| miR-30b | Late | 100/45.05% | 5.209189 | 0.02428 | 0.014392 | 0.001471 |
| miR-218 | Late | 100/56.50% | 4.616292 | 0.018837 | 0.030385 | 0.008872 |
| miR-206 | Late | 104/48.37% | 1.479038 | 0.037287 | 0.03441 | 0.001291 |
| miR-9 | Late | 157/53.04% | 1.509503 | 0.018775 | 0.036143 | 0.006808 |
| miR-124a | Late | 127/43.79% | 1.56764 | 0.020822 | 0.043852 | 0.002916 |

**(b) Significant miRNAs in developing cerebellum for their *coherent* targets using picTar target prediction**

| miRNA Name | Dev Status | Num and % of coherent genes | Log2 (P60/P7) of the miR | Ave. Log FC offset | P-val (a) | P-val (b) |
|---|---|---|---|---|---|---|
| miR-19a | Early | 131/52.19% | -2.48543 | 0.031998 | 1.14E-05 | 8.36E-10 |
| miR-106 | Early | 135/55.79% | -3.92851 | 0.032893 | 2.34E-05 | 6.78E-08 |
| miR-93 | Early | 136/51.32% | -3.27162 | 0.030942 | 0.000133 | 7.69E-09 |
| miR-144 | Early | 98/47.57% | -1.41504 | 0.030625 | 0.001148 | 0.025833 |
| miR-130 | Early | 120/57.97% | -1.65573 | 0.021509 | 0.002914 | 0.00288 |
| miR-153 | Early | 72/49.32% | -0.61005 | 0.040856 | 0.005503 | 0.003133 |
| miR-181a | Early | 86/48.04% | -0.24233 | 0.027922 | 0.005632 | 1.62E-09 |
| miR-184 | Early | 5/50% | -0.45943 | 0.12286 | 0.006003 | 0.005582 |
| miR-33 | Early | 43/56.58% | -0.96829 | 0.046944 | 0.012599 | 0.000676 |
