# Supplementary material for: Tissue and Process Specific microRNA–mRNA Co-Expression in Mammalian Development and Malignancy
Source: PLoS One. 2009 May 5;4(5):e5436. doi: 10.1371/journal.pone.0005436 (PMC2673043; doi:10.1371/journal.pone.0005436)
Supplement: Table S4 — Significant miRNAs developing lung. (0.01 MB PDF) [file pone.0005436.s005.pdf]

Supple. Table 4: Significant miRNAs for their targets' coherence or non-coherence in developing murine lung (TargetScan prediction is used)

Significant miRNAs in lung dev in terms of coherent targets					
**miRNA Name**	**[B]** coherent targets of miR Ave. Log (Expr/P1)	**[C]** non-target background genes Ave Log(Expr/P1)	**[B]-[C]**	p-val	Dev Status
**miR-9**	-0.40858751	-0.475963001	0.067375	**0.007959**	**Late**
**miR-27**	-0.39621753	-0.475963001	0.079745	**0.01019**	**Late**
**miR-125**	-0.32390459	-0.475963001	0.152058	**0.016996**	**Late**
**miR-26**	-0.34696609	-0.475963001	0.128997	**0.018557**	**Late**
**miR-15/16/195**	-0.39837816	-0.475963001	0.077585	**0.020269**	**Late**
**miR-17/20/106**	-0.35880559	-0.475963001	0.117157	**0.028752**	**Late**
**miR-221/222**	-0.32698334	-0.475963001	0.14898	**0.046226**	**Late**
**miR-199a***	0.33455485	0.528275843	-0.19372	**0.002028**	**Early**
**miR-204/211**	0.35054426	0.528275843	-0.17773	**0.014702**	**Early**

Significant miRNAs in lung dev in terms of non-coherent targets					
**miRNA Name**	**[B]** non-coherent targets of miR Ave. Log (Expr/P1)	**[C]** non-target background genes Ave Log(Expr/P1)	**[B]-[C]**	p-val	Dev Status
**miR-146**	0.72673295	0.528275843	0.198457	**0.024069**	**Late**
**miR-140**	0.7265038	0.528275843	0.198228	**0.031186**	**Late**
**miR-125**	0.40082517	0.528275843	-0.12745	**0.03391**	**Late**
**miR-34**	0.39731154	0.528275843	-0.13096	**0.043318**	**Late**
**miR-205**	-0.31139753	-0.475963001	0.164565	**0.003512**	**Early**
**miR-124**	-0.37628123	-0.475963001	0.099682	**0.007991**	**Early**
**miR-218**	-0.38952386	-0.475963001	0.086439	**0.017277**	**Early**
