# Supplementary material for: Tissue and Process Specific microRNA–mRNA Co-Expression in Mammalian Development and Malignancy
Source: PLoS One. 2009 May 5;4(5):e5436. doi: 10.1371/journal.pone.0005436 (PMC2673043; doi:10.1371/journal.pone.0005436)
Supplement: Table S5 — Enriched non-coherent GO terms for late expressed miRNAs in developing cerebellum compared with non-targets of the same term. (0.01 MB PDF) [file pone.0005436.s006.pdf]

Supple. Table 5: Enriched Late-miR-non-coherent GO terms in developing Cerebellum compared with non-targets of the same term

GO terms	Term ID	p-val (median) of non-coherent targets vs. non-miR target gene bkgd	LogFC Val (median) of non-coherent targets vs. non-miR target gene bkgd	# of miR incidences in Dev.	miR Names						
**'cell communication'**	720	0.000719	0.066217	1	**mir-138**						
**signal transduction'**	1345	0.015308	0.051985	1	**mir-138**						
**cell differentiation'**	521	0.021536	0.069223	1	**mir-9**						
**cell-cell signaling'**	912	0.006455	0.116323	6	**mir-103-1,**	**mir-128**	**mir-143**	**mir-133**	**mir-9**	**mir-138**	
**'transmission of nerve impulse'**	176	0.015832	0.160208	6	**mir-128**	**mir-133**	**mir-206**	**mir-152**	**mir-9**	**mir-138**	
**synaptic transmission'**	552	0.015832	0.160208	6	**mir-128**	**mir-133**	**mir-206**	**mir-152**	**mir-9**	**mir-138**	
**localization'**	1067	0.010958	0.061464	7	**mir-103-1,**	**mir-128**	**mir-218-1,**	**mir-23b**	**mir-21**	**mir-15**	**mir-138**
**establishment of localization'**	1253	0.01128	0.061246	7	**mir-103-1,**	**mir-128**	**mir-218-1,**	**mir-23b**	**mir-21**	**mir-15**	**mir-138**
**transport'**	869	0.012925	0.074536	2	**mir-23b**	**mir-138**					
**secretion'**	287	0.024885	0.137663	3	**mir-103-1,**	**mir-34a**	**mir-138**				
**vesicle-mediated transport'**	1527	0.00957	0.150005	1	**mir-128**						
**secretory pathway'**	1714	0.022198	0.188268	2	**mir-128**	**mir-34a**					
**exocytosis'**	1251	0.041026	0.190668	1	**mir-128**						
