# Supplementary material for: Tissue and Process Specific microRNA–mRNA Co-Expression in Mammalian Development and Malignancy
Source: PLoS One. 2009 May 5;4(5):e5436. doi: 10.1371/journal.pone.0005436 (PMC2673043; doi:10.1371/journal.pone.0005436)
Supplement: Table S6 — Enriched processes of non-coherent genes of miRNA targets common in cerebellum development and Medulloblastoma. (0.02 MB PDF) [file pone.0005436.s007.pdf]

Supple. Table 6: The enriched processes of non-coherent genes of miRNA targets common in cerebellum development and Medulloblastoma using TargetScans prediction

Synaptic Transmission Genes

*ENTREZ_*	*Gene Name*	*Corr.of two series*	*GENE_SYMBOL*	*CYTOBAND*
6506	SOLUTE CARRIER FAMILY 1 (GLIAL HIG	0.840304376	SLC1A2,	11p13-p12,
6324	SODIUM CHANNEL, VOLTAGE-GATED,	0.915801667	SCN1B,	19q13.1,
2571	GLUTAMATE DECARBOXYLASE 1 (BRA	0.95427565	GAD1,	2q31,
6857	SYNAPTOTAGMIN I	0.827120632	SYT1,	12cen-q21,
6529	SOLUTE CARRIER FAMILY 6 (NEUROTR	0.861383769	SLC6A1,	3p25-p24,
3778	POTASSIUM LARGE CONDUCTANCE CA	0.284979903	KCNMA1,	10q22.3,
777	CALCIUM CHANNEL, VOLTAGE-DEPEN	0.846770217	CACNA1E,	1q25-q31,
6616	SYNAPTOSOMAL-ASSOCIATED PROTE	0.96237188	SNAP25,	20p12-p11.2,
6535	SOLUTE CARRIER FAMILY 6 (NEUROTR	0.955618456	SLC6A8,	Xq28,
23710	GABA(A) RECEPTOR-ASSOCIATED PRO	0.908145297	GABARAPL1,	12p13.2,
4884	NEURONAL PENTRAXIN I	0.752911621	NPTX1,	17q25.1-q25.2,
3751	POTASSIUM VOLTAGE-GATED CHANNE	0.983226321	KCND2,	7q31,
1742	DISCS, LARGE HOMOLOG 4 (DROSOPH	0.670769114	DLG4,	17p13.1,
5376	PERIPHERAL MYELIN PROTEIN 22	0.946977847	PMP22,	17p12-p11.2,
2572	GLUTAMATE DECARBOXYLASE 2 (PAN	0.878199415	GAD2,	10p11.23,
1837	DYSTROBREVIN, ALPHA	0.984315547	DTNA,	18q12,
6014	RAS-LIKE WITHOUT CAAX 2	0.835018553	RIT2,	18q12.3,
3363	5-HYDROXYTRYPTAMINE (SEROTONIN	0.831016197	HTR7,	10q21-q24,

Exocytosis Genes

*ENTREZ_*	*Gene Name*	*Corr.of two series*	*GENE_SYMBOL*	*CYTOBAND*
6857	SYNAPTOTAGMIN I	0.835018553	SYT1,	12cen-q21,
6860	SYNAPTOTAGMIN IV	0.66333438	SYT4,	18q12.3,
286	ANKYRIN 1, ERYTHROCYTIC	0.986182828	ANK1,	8p11.1,
6616	SYNAPTOSOMAL-ASSOCIATED PROTE	0.984315547	SNAP25,	20p12-p11.2,

All 62 genes in the commonly shared two GO term trees (Figure 3b) between cerebellum dev and MB

*ENTREZ_*	*Gene Name*	*Corr.of two series*	*GENE_SYMBOL*	*CYTOBAND*
286	ANKYRIN 1, ERYTHROCYTIC	0.986182828	ANK1,	8p11.1,
287	ANKYRIN 2, NEURONAL	0.499512377	ANK2,	4q25-q27,
375	ADP-RIBOSYLATION FACTOR 1	0.770003861	ARF1,	1q42,
389	RAS HOMOLOG GENE FAMILY, MEMBE	0.649063954	RHOC,	1p13.1,
481	ATPASE, NA+/K+ TRANSPORTING, BET	0.720173354	ATP1B1,	1q24,
694	B-CELL TRANSLOCATION GENE 1, ANT	0.67326326	BTG1,	12q22,
777	CALCIUM CHANNEL, VOLTAGE-DEPEN	0.840304376	CACNA1E,	1q25-q31,
818	CALCIUM/CALMODULIN-DEPENDENT P	0.743515181	CAMK2G,	10q22,
867	CAS-BR-M (MURINE) ECOTROPIC RETR	0.289080968	CBL,	11q23.3,
1182	CHLORIDE CHANNEL 3	0.811213064	CLCN3,	4q33,
1399	V-CRK SARCOMA VIRUS CT10 ONCOG	0.873541364	CRKL,	22q11,
1742	DISCS, LARGE HOMOLOG 4 (DROSOPH	0.915801667	DLG4,	17p13.1,
1837	DYSTROBREVIN, ALPHA	0.95427565	DTNA,	18q12,
2046	EPH RECEPTOR A8	0.705574641	EPHA8,	1p36.12,
2562	GAMMA-AMINOBUTYRIC ACID (GABA) A	0.822395965	GABRB3,	15q11.2-q12,

2571	GLUTAMATE DECARBOXYLASE 1 (BRA	0.827120632	GAD1,	2q31,
2572	GLUTAMATE DECARBOXYLASE 2 (PAN	0.861383769	GAD2,	10p11.23,
2664	GDP DISSOCIATION INHIBITOR 1	0.881671469	GDI1,	Xq28,
2697	GAP JUNCTION PROTEIN, ALPHA 1, 43I	0.880085576	GJA1,	6q21-q23.2,
3091	HYPOXIA-INDUCIBLE FACTOR 1, ALPHA	-0.058422399	HIF1A,	14q21-q24,
3363	5-HYDROXYTRYPTAMINE (SEROTONIN	0.284979903	HTR7,	10q21-q24,
3742	POTASSIUM VOLTAGE-GATED CHANNE	0.930652545	KCNA6,	12p13,
3751	POTASSIUM VOLTAGE-GATED CHANNE	0.846770217	KCND2,	7q31,
3778	POTASSIUM LARGE CONDUCTANCE CA	0.96237188	KCNMA1,	10q22.3,
3839	KARYOPHERIN ALPHA 3 (IMPORTIN AL	0.36848981	KPNA3,	13q14.3,
3927	LIM AND SH3 PROTEIN 1	0.50113931	LASP1,	17q11-q21.3,
4035	LOW DENSITY LIPOPROTEIN-RELATED	0.830517325	LRP1,	12q13-q14,
4884	NEURONAL PENTRAXIN I	0.955618456	NPTX1,	17q25.1-q25.2,
5126	PROPROTEIN CONVERTASE SUBTILISI	0.793902945	PCSK2,	20p11.2,
5376	PERIPHERAL MYELIN PROTEIN 22	0.908145297	PMP22,	17p12-p11.2,
5861	RAB1A, MEMBER RAS ONCOGENE FAM	0.813275298	RAB1A,	2p14,
5870	RAB6A, MEMBER RAS ONCOGENE FAM	0.381956075	RAB6A,	11q13.3,
6014	RAS-LIKE WITHOUT CAAX 2	0.752911621	RIT2,	18q12.3,
6195	RIBOSOMAL PROTEIN S6 KINASE, 90KD	0.972866082	RPS6KA1,	1p,
6324	SODIUM CHANNEL, VOLTAGE-GATED, T	0.983226321	SCN1B,	19q13.1,
6334	SODIUM CHANNEL, VOLTAGE GATED, T	0.884108207	SCN8A,	12q13,
6456	SH3-DOMAIN GRB2-LIKE 2	0.893789077	SH3GL2,	9p22,
6506	SOLUTE CARRIER FAMILY 1 (GLIAL HIG	0.670769114	SLC1A2,	11p13-p12,
6529	SOLUTE CARRIER FAMILY 6 (NEUROTR	0.946977847	SLC6A1,	3p25-p24,
6535	SOLUTE CARRIER FAMILY 6 (NEUROTR	0.878199415	SLC6A8,	Xq28,
6574	SOLUTE CARRIER FAMILY 20 (PHOSPH	0.939900668	SLC20A1,	2q11-q14,
6616	SYNAPTOSOMAL-ASSOCIATED PROTE	0.984315547	SNAP25,	20p12-p11.2,
6812	SYNTAXIN BINDING PROTEIN 1	0.940864845	STXBP1,	9q34.1,
6857	SYNAPTOTAGMIN I	0.835018553	SYT1,	12cen-q21,
6860	SYNAPTOTAGMIN IV	0.66333438	SYT4,	18q12.3,
7163	TUMOR PROTEIN D52	0.935594448	TPD52,	8q21,
7881	POTASSIUM VOLTAGE-GATED CHANNE	0.897360312	KCNAB1,	3q26.1,
8648	NUCLEAR RECEPTOR COACTIVATOR 1	0.893530357	NCOA1,	2p23,
8674	VESICLE-ASSOCIATED MEMBRANE PR	0.801066838	VAMP4,	1q24-q25,
8899	PRP4 PRE-MRNA PROCESSING FACTO	0.852219456	PRPF4B,	6p25.2,
8997	KALIRIN, RHOGEF KINASE	0.943365465	KALRN,	3q21.1-q21.2, 3
9475	RHO-ASSOCIATED, COILED-COIL CONT	0.929244648	ROCK2,	2p24,
9900	SYNAPTIC VESICLE GLYCOPROTEIN 2A	0.928978401	SV2A,	1q21.2,
9962	SOLUTE CARRIER FAMILY 23 (NUCLEO	0.708633627	SLC23A2,	20p13,
10099	TETRASPANIN 3	0.815377513	TSPAN3,	15q24.3,
10580	SORBIN AND SH3 DOMAIN CONTAINING	0.707201356	SORBS1,	10q23.3-q24.1,
10776	CYCLIC AMP PHOSPHOPROTEIN, 19 KD	0.91204233	ARPP-19,	15q21.2,
23710	GABA(A) RECEPTOR-ASSOCIATED PRO	0.831016197	GABARAPL1,	12p13.2,
29993	PROTEIN KINASE C AND CASEIN KINAS	0.886885064	PACSIN1,	6p21.3,
50650	RHO GUANINE NUCLEOTIDE EXCHANG	0.950115294	ARHGEF3,	3p21-p13,
54793	POTASSIUM CHANNEL TETRAMERISAT	0.923816164	KCTD9,	8p21.1,
55861	CHROMOSOME 20 OPEN READING FRA	0.975399971	C20orf35,	20q13.12,

3

3q21.2,
