# Supplementary material for: Tissue and Process Specific microRNA–mRNA Co-Expression in Mammalian Development and Malignancy
Source: PLoS One. 2009 May 5;4(5):e5436. doi: 10.1371/journal.pone.0005436 (PMC2673043; doi:10.1371/journal.pone.0005436)
Supplement: Table S8 — Significant GO terms in developing cerebellum comparing targets with non-target control set (PITA target prediction is used). (0.01 MB PDF) [file pone.0005436.s009.pdf]

Supple. Table 8: Significant GO terms in developing cerebellum comparing targets with non-target control set (PITA target prediction is used)

		Gene Ontological term	p	off set value of logFC from control sets	# of miRNAs	
Non-coherent terms	sort by multiplicity of miRs	**'transmission of nerve impulse'**	0.00196	0.15073452	13	mir-125,-128,-137,-139,-140,-146,-218,-27b,-22,-133,-223,-152,-9
		**'synaptic transmission'**	0.00206	0.16730586	12	mir-125,-128,-139,-140,-146,-218,-27b,-22,-133,-223,-152,-9
		**'transport'**	0.00043	0.0848964	12	mir-103,-128,-137,-140,-218,-23b,-27b,-101,-223,-15,-9,-138
		**'synapse'**	0.00145	0.20002359	11	mir-128,-140,-146,-218,-27b,-34,-22,-133,-206,-15,-152
		**'cell junction'**	0.00163	0.15601536	11	mir-128,-146,-218,-27b,-30b,-34,-22,-133,-206,-152,-9
	sort by p-vals	**'binding'**	3.3E-06	0.0455292	1	mir-218
		**'cell'**	7.6E-06	0.04341264	1	mir-218
		**'phosphoprotein'**	2.2E-05	0.05780538	1	mir-128
		**'cell communication'**	2.9E-05	0.06087646	3	mir-128,-139,-138
		**'localization'**	3E-05	0.08478973	8	mir-128,-140,-218,-23b,-101,-223,-15,-138
	sort by offset from non-targets	**'membrane trafficking'**	0.003	0.35049498	1	mir-27b
		**'clathrin-coated vesicle'**	0.00438	0.29631358	2	mir-27b,-133
		**'microtubule associated complex'**	0.00385	0.26621456	1	mir-26
		**'cytoplasmic membrane-bound vesicle'**	0.0045	0.28686995	3	mir-23b,-34,-133
		**'coated vesicle'**	0.00438	0.29631358	2	mir-27b,-133
Coherent terms	sort by multiplicity of miRs	**'transport'**	0.00195	0.1022341	6	mir-122a,-153,-219,-33,-144,-106
		**'establishment of localization'**	0.00089	0.09291339	6	mir-122a,-153,-219,-33,-144,-106
		**'cytoplasmic vesicle'**	0.01047	0.11882022	5	mir-153,-19,-93,-130,-106
		**'cell junction'**	0.01509	0.1605537	5	mir-122a,-153,-181,-19,-217
		**'vesicle'**	0.01115	0.10509763	4	mir-153,-19,-93,-106
	sort by p-vals	**'binding'**	1E-05	0.04122201	1	mir-106
		**'cell'**	5.5E-05	0.0421842	1	mir-106
		**'plasma membrane'**	2.6E-05	0.1128881	1	mir-153
		**'intracellular organelle'**	0.00032	0.04619471	1	mir-106
		**'purine nucleotide binding'**	0.00028	0.11273457	1	mir-219
	sort by offset from non-targets	**'exocytosis'**	0.00293	0.34902873	1	mir-153
		**'regulation of transport'**	0.00312	0.33001786	1	mir-153
		**'secretory pathway'**	0.00102	0.294373	1	mir-153
		**'cytoplasmic membrane-bound vesicle'**	0.00328	0.23523918	1	mir-153
		**'secretion'**	0.00021	0.25211073	1	mir-153

mir-9	
	0
	0
0	0
0	0
0	0
0	0
0	0
0	0
0	0
0	0
0	0
0	0
0	0
0	0
	0
	0
0	0
0	0
0	0
0	0
0	0
0	0
0	0
0	0
0	0
0	0
0	0
0	0
0	0
