# Supplementary material for: Tissue and Process Specific microRNA–mRNA Co-Expression in Mammalian Development and Malignancy
Source: PLoS One. 2009 May 5;4(5):e5436. doi: 10.1371/journal.pone.0005436 (PMC2673043; doi:10.1371/journal.pone.0005436)
Supplement: Table S9 — Significant GO terms in developing cerebellum comparing targets with non-target control set (picTar target prediction is used). (0.01 MB PDF) [file pone.0005436.s010.pdf]

Supple. Table 9: Significant GO terms in developing cerebellum comparing targets with non-target control set (picTar target prediction is used)

		Gene Ontological term	p	off set value of logFC from control sets	# of miRNAs	
Non-coherent terms	sort by multiplicity of miRs	'cell junction'	0.006236	0.151849913	11	mir-125,-128,-137,-146,-218,-23b,-27b,-34,-22,-133,-206
		'synapse'	0.006528	0.208185125	8	mir-128,-137,-146,-218,-27b,-34,-22,-206
		'transmission of nerve impulse'	0.000855	0.1603155	7	mir-128,-27b,-34,-133,-206,-152,-9
		'synaptic transmission'	0.00098	0.178864985	7	mir-128,-27b,-34,-133,-206,-152,-9
		'cell projection'	0.007125	0.13770687	7	mir-103,-128,-218,-101,-206,-15, let-7a
	sort by p-vals	'binding'	2.25E-05	0.042488943	1	mir-103
		'cell'	2.4E-05	0.0437635	1	mir-103
		'protein binding'	2.78E-05	0.048050004	2	mir-103,-15
		'transport'	6.31E-05	0.091034972	4	mir-103,-218,-101,-15
		'establishment of localization'	0.000176	0.075996489	6	mir-103,-128,-218,-101,-133,-15
	sort by offset from non-targets	'ion channel activity'	0.007935	0.314467125	1	mir-15
		'SNARE binding'	0.000791	0.311694225	1	mir-206
		'neurotransmitter secretion'	0.00459	0.297756425	4	mir-128,-34,-133,-206
		'regulation of neurotransmitter levels'	0.001633	0.285322648	4	mir-128,-34,-133,-206
		'clathrin-coated vesicle'	0.011711	0.27584591	1	mir-133
Coherent terms	sort by multiplicity of miRs	'membrane'	0.000366	0.06658696	8	mir-122,-19,-33,-93,-144,-130,-106,-184
		'synaptic transmission'	0.011475	0.108695394	7	mir-153,-30b,-93,-92,-130,-106,-7
		'transport'	0.00015	0.084563773	6	mir-153,-33,-144,-92,-130,-106
		'transmission of nerve impulse'	0.00418	0.12694566	5	mir-153,-19,-93,-130,-106
		'establishment of localization'	9.23E-05	0.082293521	5	mir-153,-33,-144,-92,-130
	sort by p-vals	'establishment of localization'	9.23E-05	0.082293521	5	mir-153,-33,-144,-92,-130
		'vesicle-mediated transport'	0.00012	0.146750035	1	mir-153
		'transport'	0.00015	0.084563773	6	mir-153,-33,-144,-92,-130,-106
		'localization'	0.000226	0.074940921	4	mir-153,-144,-92,-130
		'nervous system development'	0.000246	0.10007885	1	mir-144
	sort by offset from non-targets	'disease mutation'	0.03234	0.28356959	1	mir-216
		'membrane trafficking'	0.015462	0.301388125	1	mir-130
		'SNARE binding'	0.003974	0.303298915	1	mir-153
		'regulation of transport'	0.005598	0.264741285	1	mir-153
		'potassium ion binding'	0.004451	0.249314015	1	mir-144
