# Supplementary material for: Tissue and Process Specific microRNA–mRNA Co-Expression in Mammalian Development and Malignancy
Source: PLoS One. 2009 May 5;4(5):e5436. doi: 10.1371/journal.pone.0005436 (PMC2673043; doi:10.1371/journal.pone.0005436)
Supplement: Table S10 — Summary of the enriched GO terms of late-expressed miRNA coherent/non-coherent targets in murine Ptch+/− MB and human MB cell line. (0.03 MB PDF) [file pone.0005436.s011.pdf]

Supple. Table 10: Summary of the enriched GO terms of miRNA coherent/non-coherent targets in murine Ptch+/- MB and human MB cell line

Up regulated in Ptch+/- MB Late coherent

GO terms	p-val (average) of coherent targets vs. non-miR target gene bkgd	LogFC Val (average) of coherent targets vs. non-miR target gene bkgd	# of miRs	miRs
'mRNA processing'	0.00591916	0.10080124	1	mir-206
'mRNA metabolism'	0.00591916	0.10080124	1	mir-206
'IPR012677:Nucleotide-binding, alpha-beta pl	0.00693191	0.10340236	1	mir-206
'IPR000504:RNA-binding region RNP-1 (RNA	0.00693191	0.10340236	1	mir-206
'SM00360:RRM'	0.00693191	0.10340236	1	mir-206
'mutagenesis site'	0.00859408	0.15509811	1	mir-206
'RNA processing'	0.00880291	0.08460562	1	mir-206
'biopolymer metabolism'	0.00977359	0.05594285	1	mir-206
'RNA metabolism'	0.00880291	0.08460562	1	mir-206
'mrna processing'	0.02112273	0.09456236	1	mir-194
'RNA splicing, via transesterification reactions	0.02112273	0.09456236	1	mir-194
'RNA splicing, via transesterification reactions	0.02112273	0.09456236	1	mir-194
'cell cycle'	0.01816385	0.12216898	1	mir-182
'nuclear mRNA splicing, via spliceosome'	0.02112273	0.09456236	1	mir-194
'mrna splicing'	0.02112273	0.09456236	1	mir-194

Down regulated in Ptch+/- MB Late Non-coherent

GO terms	p-val (average) of non-coherent targets vs. non-miR target gene bkgd	LogFC Val (average) of non-coherent targets vs. non-miR target gene bkgd	# of miRs	miRs										
'transmission of nerve impulse'	0.00425142	-0.19440714	8	mir-128	mir-146	mir-218	mir-133	mir-206	mir-152	mir-9	mir-138	0	0	0
'synaptic transmission'	0.00425142	-0.19440714	8	mir-128	mir-146	mir-218	mir-133	mir-206	mir-152	mir-9	mir-138	0	0	0
'transporter activity'	0.00679501	-0.11601993	8	mir-128	mir-137	mir-218	mir-221	mir-133	mir-206	mir-152	mir-138	0	0	0
'transport'	0.00076535	-0.10506485	5	mir-128	mir-137	mir-218	mir-133	mir-138	0	0	0	0	0	0

'cell'	0.00191215	-0.05576392	5	mir-103-1,	mir-146	mir-218	mir-133	mir-138	0	0	0	0	0	0
'cation transporter activity'	0.01374997	-0.17248573	4	mir-218	mir-133	mir-206	mir-138	0	0	0	0	0	0	0
'metal ion-binding site:Calcium 2'	0.0044212	-0.24100149	4	mir-128	mir-34a	mir-152	let-7a	0	0	0	0	0	0	0
'metal ion-binding site:Calcium 1'	0.0044212	-0.24100149	4	mir-128	mir-34a	mir-152	let-7a	0	0	0	0	0	0	0
'cell communication'	0.00285472	-0.09453811	4	mir-146	mir-218	mir-133	mir-138	0	0	0	0	0	0	0
'cell-cell signaling'	0.00150756	-0.19637578	4	mir-128	mir-218	mir-133	mir-206	0	0	0	0	0	0	0
'localization'	0.00037832	-0.10459034	4	mir-128	mir-137	mir-218	mir-133	0	0	0	0	0	0	0
'ion transporter activity'	0.00482525	-0.13967046	4	mir-128	mir-218	mir-206	mir-138	0	0	0	0	0	0	0
'transmembrane'	0.0039447	-0.08628531	4	mir-218	mir-133	mir-223	mir-138	0	0	0	0	0	0	0
'synapse'	0.00670667	-0.25584708	4	mir-146	mir-34a	mir-15	mir-124a	0	0	0	0	0	0	0
'establishment of localization'	0.00037832	-0.10459034	4	mir-128	mir-137	mir-218	mir-133	0	0	0	0	0	0	0
'physiological process'	0.00568877	-0.06188333	4	mir-103-1,	mir-146	mir-133	mir-223	0	0	0	0	0	0	0
'cellular physiological process'	0.0019576	-0.06926669	3	mir-146	mir-133	mir-223	0	0	0	0	0	0	0	0
'secretion'	0.00814221	-0.16365501	2	mir-103-1,	mir-128	0	0	0	0	0	0	0	0	0
'signal transducer activity'	0.01631368	-0.18019698	2	mir-146	mir-221	0	0	0	0	0	0	0	0	0
'intrinsic to plasma membrane'	0.00496051	-0.13928748	2	mir-218	mir-221	0	0	0	0	0	0	0	0	0
'metal ion-binding site:Calcium 1 (via carbonyl	0.00427386	-0.26839586	2	mir-128	mir-34a	0	0	0	0	0	0	0	0	0
'integral to plasma membrane'	0.00496051	-0.13928748	2	mir-218	mir-221	0	0	0	0	0	0	0	0	0
'splice variant'	0.00801046	-0.10102988	2	mir-146	mir-218	0	0	0	0	0	0	0	0	0
'intracellular'	0.00713566	-0.08105717	2	mir-103-1,	mir-146	0	0	0	0	0	0	0	0	0
'vesicle-mediated transport'	0.00912386	-0.1636569	2	mir-103-1,	mir-128	0	0	0	0	0	0	0	0	0
'transmembrane protein'	0.00640711	-0.10964766	2	mir-218	mir-133	0	0	0	0	0	0	0	0	0
'calcium ion binding'	0.00435977	-0.13501175	2	mir-34a	mir-124a	0	0	0	0	0	0	0	0	0
'cellular process'	0.00045367	-0.09905788	2	mir-146	mir-133	0	0	0	0	0	0	0	0	0
'cation binding'	0.01039352	-0.07091893	1	mir-34a	0	0	0	0	0	0	0	0	0	0
'development'	0.00342997	-0.08442168	1	mir-139	0	0	0	0	0	0	0	0	0	0
'catalytic activity'	0.00849288	-0.05063566	1	mir-133	0	0	0	0	0	0	0	0	0	0
'vesicle'	0.00187468	-0.27951036	1	mir-128	0	0	0	0	0	0	0	0	0	0
'alkali metal ion binding'	0.00582689	-0.24387737	1	mir-218	0	0	0	0	0	0	0	0	0	0
'channel or pore class transporter activity'	0.00996371	-0.23115792	1	mir-206	0	0	0	0	0	0	0	0	0	0
'binding'	0.00146769	-0.13541437	1	mir-146	0	0	0	0	0	0	0	0	0	0
'negative regulation of cellular process'	0.00684582	-0.06897961	1	mir-124a	0	0	0	0	0	0	0	0	0	0
'membrane-bound vesicle'	0.00187468	-0.27951036	1	mir-128	0	0	0	0	0	0	0	0	0	0
'protein complex assembly'	0.0028159	-0.18399679	1	mir-206	0	0	0	0	0	0	0	0	0	0
'negative regulation of cellular physiological pr	0.00684582	-0.06897961	1	mir-124a	0	0	0	0	0	0	0	0	0	0
'protein'	0.00366672	-0.12766909	1	mir-223	0	0	0	0	0	0	0	0	0	0
'alpha-type channel activity'	0.00996371	-0.23115792	1	mir-206	0	0	0	0	0	0	0	0	0	0

'regulation of neurotransmitter levels'	0.00134684	-0.24048096	1	mir-128	0	0	0	0	0	0	0	0	0	0
'membrane'	0.00582189	-0.07041844	1	mir-133	0	0	0	0	0	0	0	0	0	0
'multigene family'	0.00411201	-0.34378219	1	mir-146	0	0	0	0	0	0	0	0	0	0
'exocytosis'	0.00096199	-0.23542456	1	mir-128	0	0	0	0	0	0	0	0	0	0
'negative regulation of physiological process'	0.00684582	-0.06897961	1	mir-124a	0	0	0	0	0	0	0	0	0	0
'ion transport'	0.01356637	-0.15587877	1	mir-206	0	0	0	0	0	0	0	0	0	0
'secretory pathway'	0.00096199	-0.23542456	1	mir-128	0	0	0	0	0	0	0	0	0	0
'monovalent inorganic cation transport'	0.02608799	-0.18576565	1	mir-206	0	0	0	0	0	0	0	0	0	0
'muscle contraction'	0.01188359	-0.15732593	1	mir-30b	0	0	0	0	0	0	0	0	0	0

Down regulated in Ptch+/- MB Early coherent

GO terms	p-val (average) of coherent targets vs. non-miR target gene bkgd	LogFC Val (average) of coherent targets vs. non-miR target gene bkgd	# of miRs	miRs										
'localization'	0.00172503	-0.09300527	3	mir-153	mir-33	mir-92-1	0	0	0	0	0	0	0	0
'establishment of localization'	0.00172503	-0.09300527	3	mir-153	mir-33	mir-92-1	0	0	0	0	0	0	0	0
'transmission of nerve impulse'	0.0130364	-0.15202731	2	mir-153	mir-19a	0	0	0	0	0	0	0	0	0
'synaptic transmission'	0.0130364	-0.15202731	2	mir-153	mir-19a	0	0	0	0	0	0	0	0	0
'transport'	0.0036384	-0.09540009	2	mir-33	mir-92-1	0	0	0	0	0	0	0	0	0
'cell'	0.00361105	-0.04501405	2	mir-153	mir-92-1	0	0	0	0	0	0	0	0	0
'secretion'	0.00757369	-0.14844736	1	mir-153	0	0	0	0	0	0	0	0	0	0
'cell-cell signaling'	0.0021271	-0.14268852	1	mir-153	0	0	0	0	0	0	0	0	0	0
'membrane fraction'	0.00394401	-0.14684528	1	mir-92-1,2	0	0	0	0	0	0	0	0	0	0
'ion transporter activity'	0.01442771	-0.17552556	1	mir-181a	0	0	0	0	0	0	0	0	0	0
'synapse'	0.00636344	-0.18120322	1	mir-153	0	0	0	0	0	0	0	0	0	0
'lipoprotein'	0.01366499	-0.12741185	1	mir-153	0	0	0	0	0	0	0	0	0	0
'ion transport'	0.01442771	-0.17552556	1	mir-181a	0	0	0	0	0	0	0	0	0	0

Up regulated in Ptch+/- MB Early non-coherent

GO terms	p-val (average) of non-coherent targets vs. non-miR target gene bkgd	LogFC Val (average) of non-coherent targets vs. non-miR target gene bkgd	# of miRs	miRs

'regulation of cellular process'	0.0007866	0.08524176	1	mir-93
'regulation of biological process'	0.0007866	0.08524176	1	mir-93
'regulation of physiological process'	0.0011629	0.08584927	1	mir-93
'regulation of cellular physiological process'	0.0011629	0.08584927	1	mir-93
'regulation of metabolism'	0.00654069	0.07724448	1	mir-93
'regulation of nucleobase, nucleoside, nucleot	0.00461675	0.08064551	1	mir-93
'regulation of cellular metabolism'	0.00654069	0.07724448	1	mir-93
'cell cycle'	0.01341154	0.13896142	1	mir-33
'homeobox'	0.01693654	0.12604467	1	mir-199a-1
'IPR001356:Homeobox'	0.01693654	0.12604467	1	mir-199a-1
'SM00389:HOX'	0.01693654	0.12604467	1	mir-199a-1
'IPR012287:Homeodomain-related'	0.01693654	0.12604467	1	mir-199a-1

Up regulated in Human MB cell line Late coherent

GO terms	p-val (average) of coherent targets vs. non-miR target gene bkgd	LogFC Val (average) of coherent targets vs. non-miR target gene bkgd	[illegible] miR incidences in human MB cancer cell line.	miRs										
'signal'	0.00104444	1.56655737	7	mir-23b	mir-26a	mir-34a	mir-206	let-7a	mir-124a	mir-9	0	0	0	0
'HSA04510:FOCAL ADHESION'	0.0108423	1.52465622	5	mir-128	mir-30b	let-7a	mir-124a	mir-9	0	0	0	0	0	0
'extracellular matrix (sensu Metazoa)'	0.00322726	2.53287098	4	mir-26a	let-7a	mir-124a	mir-9	0	0	0	0	0	0	0
'development'	0.01253824	1.50059951	4	mir-128	mir-23b	mir-199a	mir-124a	0	0	0	0	0	0	0
'structural protein'	0.00344947	1.99684027	4	mir-34a	let-7a	mir-124a	mir-9	0	0	0	0	0	0	0
'extracellular matrix'	0.00395093	2.53978239	4	mir-26a	let-7a	mir-124a	mir-9	0	0	0	0	0	0	0
'IPR008160:Collagen triple helix repeat'	0.00498399	3.14101186	4	mir-26a	let-7a	mir-124a	mir-9	0	0	0	0	0	0	0
'phosphate transport'	0.00498399	3.14101186	4	mir-26a	let-7a	mir-124a	mir-9	0	0	0	0	0	0	0
'HSA04512:ECM-RECEPTOR INTERACTION	0.00964291	2.93395567	4	mir-128	mir-26a	let-7a	mir-124a	0	0	0	0	0	0	0
'anion transport'	0.00519097	2.79271476	4	mir-26a	let-7a	mir-124a	mir-9	0	0	0	0	0	0	0
'inorganic anion transport'	0.00498399	3.14101186	4	mir-26a	let-7a	mir-124a	mir-9	0	0	0	0	0	0	0
'collagen'	0.00812781	2.9945617	4	mir-26a	let-7a	mir-124a	mir-9	0	0	0	0	0	0	0
'hydroxylation'	0.0051186	2.99596402	4	mir-26a	let-7a	mir-124a	mir-9	0	0	0	0	0	0	0
'cellular morphogenesis'	0.00725582	2.2411928	3	mir-34a	mir-96	mir-9	0	0	0	0	0	0	0	0
'cell proliferation'	0.01244947	1.47920647	3	mir-139	mir-96	mir-124a	0	0	0	0	0	0	0	0
'skeletal development'	0.01474525	2.52060448	3	mir-26a	mir-124a	mir-9	0	0	0	0	0	0	0	0
'nucleotide phosphate-binding region:ATP'	0.01123352	1.2256117	3	mir-103-1,	mir-128	mir-30b	0	0	0	0	0	0	0	0

'regulation of cell proliferation'	0.01385384	1.37501312	3	mir-139	mir-124a	mir-9	0	0	0	0	0	0	0	0
'transmembrane protein'	0.00691429	2.56896458	3	mir-23b	mir-34a	mir-223	0	0	0	0	0	0	0	0
'glycoprotein'	7.4385E-05	1.92983142	3	mir-23b	mir-34a	let-7a	0	0	0	0	0	0	0	0
'direct protein sequencing'	0.00939947	1.67104818	2	let-7a	mir-9	0	0	0	0	0	0	0	0	0
'purine nucleotide binding'	0.01386792	1.43489503	2	mir-128	let-7a	0	0	0	0	0	0	0	0	0
'HSA01430:CELL COMMUNICATION'	0.00183965	3.15917646	2	let-7a	mir-124a	0	0	0	0	0	0	0	0	0
'plasma membrane'	0.00471078	1.55833084	2	mir-34a	let-7a	0	0	0	0	0	0	0	0	0
'nucleotide-binding'	0.01344244	1.53988864	2	mir-128	let-7a	0	0	0	0	0	0	0	0	0
'cell adhesion'	0.00960838	1.20133505	2	mir-26a	mir-124a	0	0	0	0	0	0	0	0	0
'transferase'	0.01470168	0.69432209	2	mir-125a	mir-128	0	0	0	0	0	0	0	0	0
'cytoplasm'	0.00842129	1.10646956	2	mir-128	let-7a	0	0	0	0	0	0	0	0	0
'transport'	0.00884536	0.4833216	2	mir-92-1,2	let-7a	0	0	0	0	0	0	0	0	0
'extracellular matrix structural constituent'	0.00214038	3.78303047	2	mir-26a	let-7a	0	0	0	0	0	0	0	0	0
'coiled coil'	0.02020721	1.07370575	2	mir-103-1,	let-7a	0	0	0	0	0	0	0	0	0
'positive regulation of cellular process'	0.01200344	1.833014	2	mir-128	mir-96	0	0	0	0	0	0	0	0	0
'organ development'	0.01059971	1.63377131	2	mir-23b	mir-9	0	0	0	0	0	0	0	0	0
'IPR013320:Concanavalin A-like lectin/glucan	0.0175274	2.53916393	2	mir-26a	mir-124a	0	0	0	0	0	0	0	0	0
'duplication'	0.02264578	1.40597771	2	mir-30b	mir-124a	0	0	0	0	0	0	0	0	0
'transferase activity, transferring phosphorus-(	0.00622796	2.14875998	2	mir-128	mir-34a	0	0	0	0	0	0	0	0	0
'calcium binding'	0.03984811	1.64405962	2	mir-23b	mir-152	0	0	0	0	0	0	0	0	0
'cell organization and biogenesis'	0.02545904	0.19406755	2	mir-34a	mir-106	0	0	0	0	0	0	0	0	0
'calcium'	0.02967447	1.18967267	1	mir-152	0	0	0	0	0	0	0	0	0	0
'atp-binding'	0.00539903	1.7119972	1	mir-128	0	0	0	0	0	0	0	0	0	0
'IPR001245:Tyrosine protein kinase'	0.01157056	2.7164259	1	mir-128	0	0	0	0	0	0	0	0	0	0
'IPR006209:EGF-like'	0.01023773	2.32988007	1	mir-23b	0	0	0	0	0	0	0	0	0	0
'hydroxylysine'	0.0012657	3.04731255	1	let-7a	0	0	0	0	0	0	0	0	0	0
'developmental protein'	0.00331987	2.25031695	1	mir-23b	0	0	0	0	0	0	0	0	0	0
'hydroxyproline'	0.0012657	3.04731255	1	let-7a	0	0	0	0	0	0	0	0	0	0

Down regulated in Human MB cell line Late Non-coherent

GO terms	p-val (average) of non-coherent targets vs. non-miR-target gene bkgd	LogFC Val (average) of non-coherent targets vs. non-miR target gene bkgd	# of miR incidences in human MB cancer cell line	miRs										
'cell communication'	0.00293296	-1.5267677	15	mir-103-1,	mir-128	mir-140	mir-146	mir-181a	mir-218	mir-34a	mir-143	mir-145	mir-133	mir-206

'transmission of nerve impulse'	0.00614581	-3.72827775	14	mir-128	mir-140	mir-146	mir-218	mir-27b	mir-30b	mir-143	mir-133	mir-206	mir-15	mir-152
'synaptic transmission'	0.00614581	-3.72827775	14	mir-128	mir-140	mir-146	mir-218	mir-27b	mir-30b	mir-143	mir-133	mir-206	mir-15	mir-152
'transport'	0.00870999	-2.18766785	11	mir-128	mir-139	mir-140	mir-146	mir-181a	mir-218	mir-34a	mir-133	mir-15	let-7a	mir-138
'cell-cell signaling'	0.00620298	-3.76080007	11	mir-128	mir-140	mir-146	mir-218	mir-27b	mir-143	mir-133	mir-206	mir-182	mir-15	mir-138
'calcium ion binding'	0.01333406	-1.66795127	11	mir-103-1,	mir-128	mir-19a	mir-23b	mir-26a	mir-30b	mir-133	mir-182	mir-15	mir-152	let-7a
'binding'	0.00750254	-0.84400892	10	mir-103-1,	mir-139	mir-146	mir-204	mir-26a	mir-143	mir-133	mir-223	mir-15	mir-138	0
'cellular process'	0.00886309	-0.74450199	10	mir-103-1,	mir-139	mir-146	mir-204	mir-34a	mir-145	mir-133	mir-223	mir-15	mir-138	0
'localization'	0.00652681	-1.6818742	9	mir-128	mir-139	mir-146	mir-218	mir-34a	mir-133	mir-182	let-7a	mir-138	0	0
'multigene family'	0.00482051	-1.98743317	9	mir-103-1,	mir-146	mir-218	mir-27b	mir-145	mir-133	mir-182	mir-15	mir-138	0	0
'establishment of localization'	0.00652681	-1.6818742	9	mir-128	mir-139	mir-146	mir-218	mir-34a	mir-133	mir-182	let-7a	mir-138	0	0
'protein binding'	0.01602054	-0.87251591	9	mir-103-1,	mir-139	mir-26a	mir-143	mir-145	mir-133	mir-223	mir-15	mir-138	0	0
'membrane'	0.01058195	-1.73581663	8	mir-103-1,	mir-137	mir-140	mir-146	mir-181a	mir-218	mir-206	mir-15	0	0	0
'transmembrane'	0.01601524	-1.82105656	8	mir-103-1,	mir-137	mir-140	mir-146	mir-218	mir-206	mir-15	mir-138	0	0	0
'synapse'	0.00760714	-4.05994793	7	mir-137	mir-146	mir-181a	mir-34a	mir-206	mir-15	mir-152	0	0	0	0
'cell'	0.00733566	-0.82209638	7	mir-103-1,	mir-146	mir-181a	mir-34a	mir-223	mir-15	mir-138	0	0	0	0
'calcium'	0.01297821	-2.2912011	6	mir-128	mir-26a	mir-133	mir-15	mir-152	let-7a	0	0	0	0	0
'signal transduction'	0.01449878	-1.16312001	6	mir-103-1,	mir-181a	mir-143	mir-15	mir-152	mir-138	0	0	0	0	0
'system development'	0.01750693	-2.86665653	6	mir-204	mir-133	mir-206	mir-15	mir-106	mir-138	0	0	0	0	0
'cellular physiological process'	0.00606685	-0.84661847	6	mir-103-1,	mir-139	mir-146	mir-223	mir-15	mir-138	0	0	0	0	0
'physiological process'	0.00280223	-0.86373828	6	mir-103-1,	mir-139	mir-146	mir-223	mir-15	mir-138	0	0	0	0	0
'nervous system development'	0.01750693	-2.86665653	6	mir-204	mir-133	mir-206	mir-15	mir-106	mir-138	0	0	0	0	0
'glycoprotein'	0.01012203	-2.00429264	6	mir-218	mir-221	mir-133	mir-206	mir-15	mir-138	0	0	0	0	0
'alternative splicing'	0.01380315	-0.92394519	6	mir-103-1,	mir-139	mir-146	mir-218	mir-15	mir-138	0	0	0	0	0
'cation transporter activity'	0.01653032	-2.92655552	5	mir-218	mir-30b	mir-182	mir-15	let-7a	0	0	0	0	0	0
'metal ion-binding site:Calcium 2'	0.00413625	-5.83548523	5	mir-128	mir-34a	mir-152	let-7a	mir-9	0	0	0	0	0	0
'metal ion-binding site:Calcium 1'	0.00413625	-5.83548523	5	mir-128	mir-34a	mir-152	let-7a	mir-9	0	0	0	0	0	0
'transporter activity'	0.01979358	-2.2709792	5	mir-137	mir-146	mir-218	mir-34a	let-7a	0	0	0	0	0	0
'splice variant'	0.01336658	-1.04873221	5	mir-103-1,	mir-139	mir-146	mir-15	mir-138	0	0	0	0	0	0
'ion binding'	0.00428612	-1.24633567	4	mir-30b	mir-34a	mir-15	let-7a	0	0	0	0	0	0	0
'cation binding'	0.00312794	-1.31579403	4	mir-30b	mir-34a	mir-15	let-7a	0	0	0	0	0	0	0
'metal ion binding'	0.00428612	-1.24633567	4	mir-30b	mir-34a	mir-15	let-7a	0	0	0	0	0	0	0
'nucleotide binding'	0.02013924	-1.1819519	4	mir-103-1,	mir-139	mir-218	mir-375	0	0	0	0	0	0	0
'cytoplasm'	0.01415696	-1.39782094	4	mir-34a	mir-15	let-7a	mir-138	0	0	0	0	0	0	0
'coiled coil'	0.0099176	-2.18133038	4	mir-103-1,	mir-146	mir-206	mir-138	0	0	0	0	0	0	0
'cytoskeleton'	0.02346268	-1.65140412	4	mir-26a	mir-34a	mir-152	mir-9	0	0	0	0	0	0	0
'phosphorylation'	0.01514142	-1.09196186	4	mir-103-1,	mir-218	mir-26a	mir-138	0	0	0	0	0	0	0
'lipoprotein'	0.00558109	-2.98393695	4	mir-103-1,	mir-128	mir-206	mir-152	0	0	0	0	0	0	0

'Unknown'	0.00169932	-1.53197267	3	mir-103-1,	mir-218	mir-138	0	0	0	0	0	0	0	0
'hydrolase activity, acting on ester bonds'	0.02644898	-0.99754197	3	mir-103-1,	mir-145	mir-15	0	0	0	0	0	0	0	0
'secretion'	0.00480475	-4.37534893	3	mir-128	mir-137	mir-34a	0	0	0	0	0	0	0	0
'hydrolase'	0.02075074	-1.31607287	3	mir-145	mir-15	let-7a	0	0	0	0	0	0	0	0
'plasma membrane'	0.02157529	-1.3054216	3	mir-204	mir-143	mir-182	0	0	0	0	0	0	0	0
'phosphoprotein'	0.02183061	-1.86550183	3	mir-128	mir-194	mir-124a	0	0	0	0	0	0	0	0
'signal'	0.01463852	-2.7353827	3	mir-139	mir-221	mir-15	0	0	0	0	0	0	0	0
'metal ion-binding site:Calcium 1 (via carbonyl	0.00383489	-6.01343303	3	mir-128	mir-34a	mir-9	0	0	0	0	0	0	0	0
'domain:Protein kinase'	0.01400438	-1.43854382	3	mir-218	mir-182	mir-124a	0	0	0	0	0	0	0	0
'intracellular'	0.006147	-0.68184795	3	mir-103-1,	mir-146	mir-15	0	0	0	0	0	0	0	0
'vesicle-mediated transport'	0.00576745	-2.98261568	3	mir-103-1,	mir-128	mir-34a	0	0	0	0	0	0	0	0
'transmembrane protein'	0.02388183	-1.93987693	3	mir-218	mir-34a	mir-133	0	0	0	0	0	0	0	0

Down regulated in Human MB cell line Early coherent

GO terms	p-val (average) of coherent targets vs. non-miR-target gene bkgd	LogFC Val (average) of coherent targets vs. non-miR target gene bkgd	# of miR incidences in human MB cancer cell line	miRs										
'binding'	0.01549341	-0.53569936	5	mir-30b	mir-33	mir-93	mir-92-1	mir-106	0	0	0	0	0	0
'multigene family'	0.00499959	-2.00381773	5	mir-153	mir-181a	mir-19a	mir-92-1	mir-106	0	0	0	0	0	0
'calcium ion binding'	0.01303695	-2.7680117	5	mir-181a	mir-19a	mir-93	mir-92-1	mir-130	0	0	0	0	0	0
'ion binding'	0.01243451	-1.47806644	4	mir-217	mir-33	mir-93	mir-92-1	0	0	0	0	0	0	0
'calcium'	0.01552967	-2.33853525	4	mir-181a	mir-19a	mir-93	mir-130	0	0	0	0	0	0	0
'metal ion binding'	0.01243451	-1.47806644	4	mir-217	mir-33	mir-93	mir-92-1	0	0	0	0	0	0	0
'ion transporter activity'	0.01262056	-3.69570842	4	mir-181a	mir-93	mir-92-1	mir-106	0	0	0	0	0	0	0
'cellular process'	0.00567631	-0.59981944	4	mir-30b	mir-93	mir-92-1	mir-106	0	0	0	0	0	0	0
'cation binding'	0.00765852	-1.67434455	3	mir-217	mir-93	mir-92-1	0	0	0	0	0	0	0	0
'transmission of nerve impulse'	0.01046244	-3.49209867	3	mir-153	mir-19a	mir-106	0	0	0	0	0	0	0	0
'cation transporter activity'	0.01693966	-3.7219623	3	mir-93	mir-92-1	mir-106	0	0	0	0	0	0	0	0
'synaptic transmission'	0.01046244	-3.49209867	3	mir-153	mir-19a	mir-106	0	0	0	0	0	0	0	0
'cell communication'	0.01537778	-0.74981708	3	mir-181a	mir-19a	mir-33	0	0	0	0	0	0	0	0
'cell-cell signaling'	0.01046244	-3.49209867	3	mir-153	mir-19a	mir-106	0	0	0	0	0	0	0	0
'membrane'	0.01156224	-1.5990482	3	mir-122a	mir-181a	mir-92-1	0	0	0	0	0	0	0	0
'physiological process'	0.01333945	-0.64024066	3	mir-93	mir-92-1	mir-106	0	0	0	0	0	0	0	0
'development'	0.00308439	-1.98422665	2	mir-145	mir-92-1	0	0	0	0	0	0	0	0	0

'metal ion transport'	0.00890136	-4.57997693	2	mir-93	mir-106	0	0	0	0	0	0	0	0	0
'plasma membrane'	0.00929883	-1.74004929	2	mir-181a	mir-92-1	0	0	0	0	0	0	0	0	0
'signal transducer activity'	0.01096731	-1.12819614	2	mir-181a	mir-19a	0	0	0	0	0	0	0	0	0
'hydrolase activity, acting on acid anhydrides,	0.01673599	-3.22686842	2	mir-181a	mir-106	0	0	0	0	0	0	0	0	0
'phosphoprotein'	0.01167052	-1.7565378	2	mir-153	mir-92-1	0	0	0	0	0	0	0	0	0
'transmembrane'	0.02284947	-2.14994852	2	mir-122a	mir-92-1	0	0	0	0	0	0	0	0	0
'pyrophosphatase activity'	0.01673599	-3.22686842	2	mir-181a	mir-106	0	0	0	0	0	0	0	0	0
'cellular physiological process'	0.0085857	-0.56741536	2	mir-92-1,2	mir-106	0	0	0	0	0	0	0	0	0
'hydrolase activity, acting on acid anhydrides'	0.01673599	-3.22686842	2	mir-181a	mir-106	0	0	0	0	0	0	0	0	0
'ion transport'	0.00635712	-4.3330075	2	mir-181a	mir-93	0	0	0	0	0	0	0	0	0
'glycoprotein'	0.01397304	-2.58345356	2	mir-217	mir-92-1	0	0	0	0	0	0	0	0	0
'nucleoside-triphosphatase activity'	0.01673599	-3.22686842	2	mir-181a	mir-106	0	0	0	0	0	0	0	0	0
'atp-binding'	0.01716366	-1.19134701	1	mir-181a	0	0	0	0	0	0	0	0	0	0
'direct protein sequencing'	0.0219303	-2.37008343	1	mir-92-1,2	0	0	0	0	0	0	0	0	0	0
'protein metabolism'	0.02048454	-2.00288347	1	mir-122a	0	0	0	0	0	0	0	0	0	0
'HSA04020:CALCIUM SIGNALING PATHWA	0.02538249	-2.73240097	1	mir-106	0	0	0	0	0	0	0	0	0	0
'purine nucleotide binding'	0.0183162	-1.00504124	1	mir-181a	0	0	0	0	0	0	0	0	0	0
'metal ion-binding site:Magnesium'	0.00589974	-5.60311987	1	mir-106	0	0	0	0	0	0	0	0	0	0
'secretion'	0.00371753	-2.65050247	1	mir-153	0	0	0	0	0	0	0	0	0	0
'hydrolase'	0.02045386	-1.68179323	1	mir-106	0	0	0	0	0	0	0	0	0	0
'nucleotide-binding'	0.0183162	-1.00504124	1	mir-181a	0	0	0	0	0	0	0	0	0	0
'catalytic activity'	0.00174878	-1.02454625	1	mir-181a	0	0	0	0	0	0	0	0	0	0
'metabolism'	0.03445593	-0.49339676	1	mir-106	0	0	0	0	0	0	0	0	0	0
'domain:SH3'	0.02779036	-2.0247861	1	mir-27b	0	0	0	0	0	0	0	0	0	0
'cell differentiation'	0.00894551	-3.74351373	1	mir-145	0	0	0	0	0	0	0	0	0	0
'regulation of cellular process'	0.01722927	-0.97263601	1	mir-30b	0	0	0	0	0	0	0	0	0	0
'morphogenesis'	0.01904593	-1.92882805	1	mir-145	0	0	0	0	0	0	0	0	0	0
'cellular protein metabolism'	0.0241513	-0.66407273	1	mir-181a	0	0	0	0	0	0	0	0	0	0
'phosphate metabolism'	0.01869532	-0.69897807	1	mir-181a	0	0	0	0	0	0	0	0	0	0
'sh3 domain'	0.02779036	-2.0247861	1	mir-27b	0	0	0	0	0	0	0	0	0	0
'heterodimer'	0.02443352	-3.76774383	1	mir-106	0	0	0	0	0	0	0	0	0	0
'disease mutation'	0.02094698	-3.1192207	1	mir-137	0	0	0	0	0	0	0	0	0	0
'adenyl nucleotide binding'	0.01716366	-1.19134701	1	mir-181a	0	0	0	0	0	0	0	0	0	0

Up regulated in Human MB cell line Early non-coherent

GO terms	p-val (average) of non-coherent targets vs. non-miR target gene bkgd	LogFC Val (average) of non-coherent targets vs. non-miR target gene bkgd	[illegible] miR incidences in human MB cancer cell line.	miRs										
'signal'	0.00137852	1.76142661	4	mir-30b	mir-143	mir-92-1	mir-124a	0	0	0	0	0	0	0
'transcription regulation'	0.01070666	1.46394154	4	mir-217	mir-26a	mir-145	mir-144	0	0	0	0	0	0	0
'cellular process'	0.01565827	0.91535631	4	mir-139	mir-192	mir-217	mir-92-1	0	0	0	0	0	0	0
'transcription, DNA-dependent'	0.00877155	1.65247683	3	mir-217	mir-26a	mir-144	0	0	0	0	0	0	0	0
'transcription'	0.00877155	1.65247683	3	mir-217	mir-26a	mir-144	0	0	0	0	0	0	0	0
'binding'	0.01114356	0.97708029	3	mir-192	mir-217	mir-144	0	0	0	0	0	0	0	0
'intracellular organelle'	0.00986006	1.04200289	3	mir-137	mir-217	mir-144	0	0	0	0	0	0	0	0
'transcription factor activity'	0.01332337	1.629829	3	mir-26a	mir-145	mir-144	0	0	0	0	0	0	0	0
'dna-binding'	0.01210379	1.65247683	3	mir-217	mir-144	mir-92-1	0	0	0	0	0	0	0	0
'regulation of transcription, DNA-dependent'	0.00877155	1.65247683	3	mir-217	mir-26a	mir-144	0	0	0	0	0	0	0	0
'intracellular'	0.01067696	0.72663205	3	mir-137	mir-217	mir-144	0	0	0	0	0	0	0	0
'nuclear protein'	0.01491691	1.34877587	3	mir-137	mir-217	mir-144	0	0	0	0	0	0	0	0
'physiological process'	0.01326913	1.29804232	3	mir-139	mir-192	mir-217	0	0	0	0	0	0	0	0
'nucleobase, nucleoside, nucleotide and nucle	0.01677245	1.34877587	3	mir-139	mir-217	mir-144	0	0	0	0	0	0	0	0
'cell'	0.01630438	1.11376121	3	mir-192	mir-217	mir-144	0	0	0	0	0	0	0	0
'organelle'	0.00986006	1.04200289	3	mir-137	mir-217	mir-144	0	0	0	0	0	0	0	0
'glycoprotein'	0.00138727	1.14540893	3	mir-30b	mir-143	mir-124a	0	0	0	0	0	0	0	0
'regulation of transcription'	0.00877155	1.65247683	3	mir-217	mir-26a	mir-144	0	0	0	0	0	0	0	0
'regulation of physiological process'	0.00562307	2.02160447	2	mir-217	mir-143	0	0	0	0	0	0	0	0	0
'plasma membrane'	0.02133468	2.0287775	2	mir-194	mir-143	0	0	0	0	0	0	0	0	0
'membrane-bound organelle'	0.01189514	0.18930189	2	mir-137	mir-144	0	0	0	0	0	0	0	0	0
'transcription regulator activity'	0.00804731	1.56751562	2	mir-26a	mir-144	0	0	0	0	0	0	0	0	0
'cell communication'	0.01241214	0.23661282	2	mir-125a	mir-92-1	0	0	0	0	0	0	0	0	0
'nucleus'	0.02013454	1.44754192	2	mir-217	mir-144	0	0	0	0	0	0	0	0	0
'nucleic acid binding'	0.02576904	0.27066023	2	mir-139	mir-217	0	0	0	0	0	0	0	0	0
'DNA binding'	0.01043767	1.7510582	2	mir-217	mir-144	0	0	0	0	0	0	0	0	0
'regulation of biological process'	0.00562307	2.02160447	2	mir-217	mir-143	0	0	0	0	0	0	0	0	0
'regulation of nucleobase, nucleoside, nucleot	0.00865454	1.76395102	2	mir-217	mir-144	0	0	0	0	0	0	0	0	0
'intracellular membrane-bound organelle'	0.01189514	0.18930189	2	mir-137	mir-144	0	0	0	0	0	0	0	0	0
