# Supplementary material for: Tissue and Process Specific microRNA–mRNA Co-Expression in Mammalian Development and Malignancy
Source: PLoS One. 2009 May 5;4(5):e5436. doi: 10.1371/journal.pone.0005436 (PMC2673043; doi:10.1371/journal.pone.0005436)
Supplement: Table S11 — The statistic significance and other qualifications of the shared miRNA non-coherent GO terms between brain tumor and development (picTar target prediction is used). (0.01 MB PDF) [file pone.0005436.s012.pdf]

Supple. Table 11: The statistic significance and other quantifications of the shared miRNA non-coherent GO terms between brain tumor and development. (picTar target prediction is used)

Term	p-val in Dev.	LogFC Val offset in Dev.	# of miRNAs in Dev.	p-val in MB	LogFC Val offset in MB	# of miRNAs in MB	# of common genes	Significant miRNAs shared btw Ptch+/- MB and development					
**'transmission of nerve impulse'**	0.00085509	0.1603155	7	0.00179757	-0.19021323	7	20	mir-128	mir-27b	mir-133	mir-206	mir-9	
**'synaptic transmission'**	0.00097989	0.17886499	7	0.0013259	-0.19562017	8	19	mir-128	mir-27b	mir-133	mir-206	mir-152	mir-9
**'cell communication'**	0.00189153	0.07519943	2	0.0006943	-0.09264798	3	18	mir-133	0	0	0	0	
**'transport'**	6.3099E-05	0.09103497	4	0.00346232	-0.08830199	8	34	mir-103	mir-218	mir-15	0	0	
**'cell-cell signaling'**	0.00179412	0.12451218	5	0.00084774	-0.16230764	7	20	mir-128	mir-133	mir-9	0	0	
**'localization'**	0.00019289	0.06779094	6	0.00238273	-0.07947852	8	76	mir-103	mir-128	mir-133	mir-15	0	
**'establishment of localization'**	0.00017647	0.07599649	6	0.00355532	-0.08484031	9	80	mir-103	mir-128	mir-218	mir-133	mir-15	
**'exocytosis'**	0.00684165	0.2421659	4	0.00396211	-0.21921647	3	6	mir-128	mir-34	mir-206	0	0	
**'vesicle-mediated transport'**	0.00586559	-0.06710742	1	0.00586559	-0.06710742	1	13	mir-103	0	0	0	0	
